# Appraising the Causal Association between Systemic Iron Status and Heart Failure Risk: A Mendelian Randomisation Study

**DOI:** 10.3390/nu14163258

**Published:** 2022-08-09

**Authors:** Xingchen Wang, Xizhi Wang, Yingchao Gong, Xiaoou Chen, Danfeng Zhong, Jun Zhu, Lenan Zhuang, Jing Gao, Guosheng Fu, Xue Lu, Dongwu Lai

**Affiliations:** Key Laboratory of Cardiovascular Intervention and Regenerative Medicine of Zhejiang Province, Department of Cardiology, Sir Run Run Shaw Hospital, Zhejiang University School of Medicine, Hangzhou 310016, China

**Keywords:** heart failure, iron status, Mendelian randomisation, causal association

## Abstract

Although observational studies have shown that abnormal systemic iron status is associated with an increased risk of heart failure (HF), it remains unclear whether this relationship represents true causality. We aimed to explore the causal relationship between iron status and HF risk. Two-sample Mendelian randomisation (MR) was applied to obtain a causal estimate. Genetic summary statistical data for the associations (*p* < 5 × 10^−8^) between single nucleotide polymorphisms (SNPs) and four iron status parameters were obtained from the Genetics of Iron Status Consortium in genome-wide association studies involving 48,972 subjects. Statistical data on the association of SNPs with HF were extracted from the UK biobank consortium (including 1088 HF cases and 360,106 controls). The results were further tested using MR based on the Bayesian model averaging (MR-BMA) and multivariate MR (MVMR). Of the twelve SNPs considered to be valid instrumental variables, three SNPs (rs1800562, rs855791, and rs1799945) were associated with all four iron biomarkers. Genetically predicted iron status biomarkers were not causally associated with HF risk (all *p* > 0.05). Sensitivity analysis did not show evidence of potential heterogeneity and horizontal pleiotropy. Convincing evidence to support a causal relationship between iron status and HF risk was not found. The strong relationship between abnormal iron status and HF risk may be explained by an indirect mechanism.

## 1. Introduction

Heart failure (HF), a clinical syndrome of cardiac dysfunction caused by multiple factors and associated with high morbidity and poor prognosis, has become a major public health issue [1,2]. Conditions such as coronary heart disease, cardiomyopathy, and diabetes are common causes of HF. Currently, 1–2% of adults in developed countries have been diagnosed with HF [3]. Considering an ageing population, the prevalence of HF is expected to double by 2060 [4,5]. It is therefore imperative to determine the epidemiological characteristics and risk factors for the occurrence and development of HF.

Iron is involved in numerous vital biological processes, such as erythropoiesis, cellular metabolism, redox balance, and inflammation [6]. Several studies have reported that abnormal iron status is associated with various cardiovascular diseases. Meanwhile, iron deficiency (ID), as one of the most common malnutrition factors, affects approximately one-third of the worldwide population [7]. Notably, nearly half of HF patients have accompanying iron metabolism disorders (mainly ID) [8,9,10]. Further, ID is an independent predictor of poor clinical symptoms and prognosis in patients with HF [11,12,13]. In several cohort studies, both high and low serum ferritin levels were associated with increased incidence of HF [14,15]. Similarly, in animal experiments, both ID and iron overload were observed to lead to ventricular hypertrophy and abnormal cardiac function [16,17]. However, observational studies have difficulty distinguishing causal associations from spurious associations, and animal models cannot fully simulate actual clinical conditions. Therefore, it remains to be determined whether abnormal iron status is an initiating factor in HF or simply a consequence/biomarker/common comorbidity. Further studies are required to clarify whether there is a causative relationship between iron status and HF risk.

Mendelian randomisation (MR) is an emerging method of epidemiological analysis that employs genetic variants as an instrumental variable (IV) to obtain more robust causal inferences from the observed data [18,19,20]. This approach can effectively avoid the effects of reverse causality and residual confounding, thereby ensuring the robustness of the results. To date, it has not been ascertained whether systemic iron status is involved in the pathogenesis of HF in a causative manner. Consequently, this study was conducted to explore the causal association between systemic iron status biomarkers and the HF risk by employing a two-sample MR analysis.

## 2. Materials and Methods

### 2.1. Study Design and Data Source

**Figure 1:**
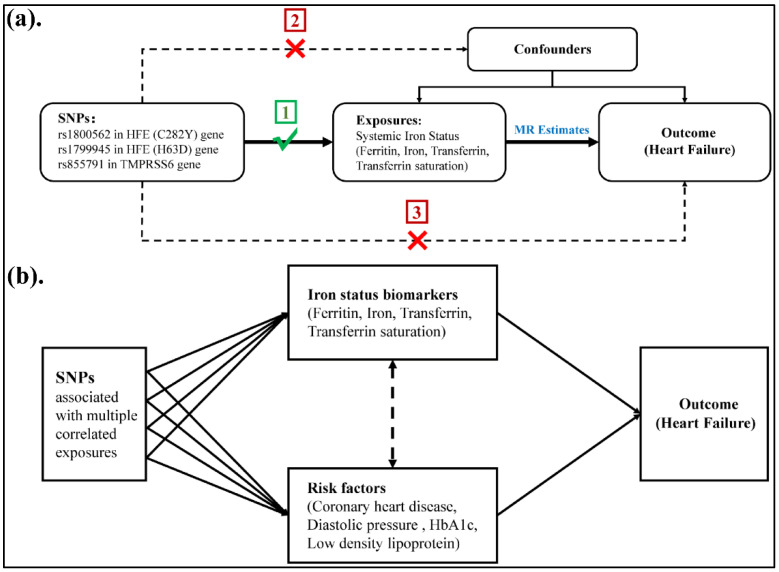
Schematic diagram of the Mendelian randomisation study design. (**a**) Two-sample Mendelian randomisation (TSMR). (**b**) Multivariate Mendelian randomisation (MVMR).

We selected genetic instruments for four iron status biomarkers: ferritin, iron, transferrin, and transferrin saturation (TS). A two-sample MR study was conducted to appraise the causal association between iron status and the relevant outcomes. The single nucleotide polymorphisms (SNPs) identified as IVs for iron status were obliged to fulfil three core premises: (1) SNPs must be robustly related to iron status biomarkers, (2) SNPs should not be related to confounders, and (3) SNPs must only be related to outcomes via iron status biomarkers (Figure 1a).

Summary statistics of SNPs related to iron status biomarkers were extracted from a previous meta-analysis of genome-wide studies conducted by the Iron Status Genetics Consortium [21]. The characteristic statistics of the patients in this meta-analysis are presented in Supplementary Table S1. Data from eleven discovery cohorts and eight replication cohorts were employed in the meta-analysis, comprising a total of 48,972 Europeans. HF data was contributed by the Neale lab analysis of UK Biobank phenotypes (http://www.nealelab.is/uk-biobank (accessed on 15 January 2022)), with a sample size of 361,194 (1405 HF cases and 359,789 controls). Characteristics and summary data of the relationship between SNPs and iron status parameters and HF are shown in Table 1 and Supplementary Table S2, respectively.

### 2.2. SNP Selection and Validation

**Figure 2:**
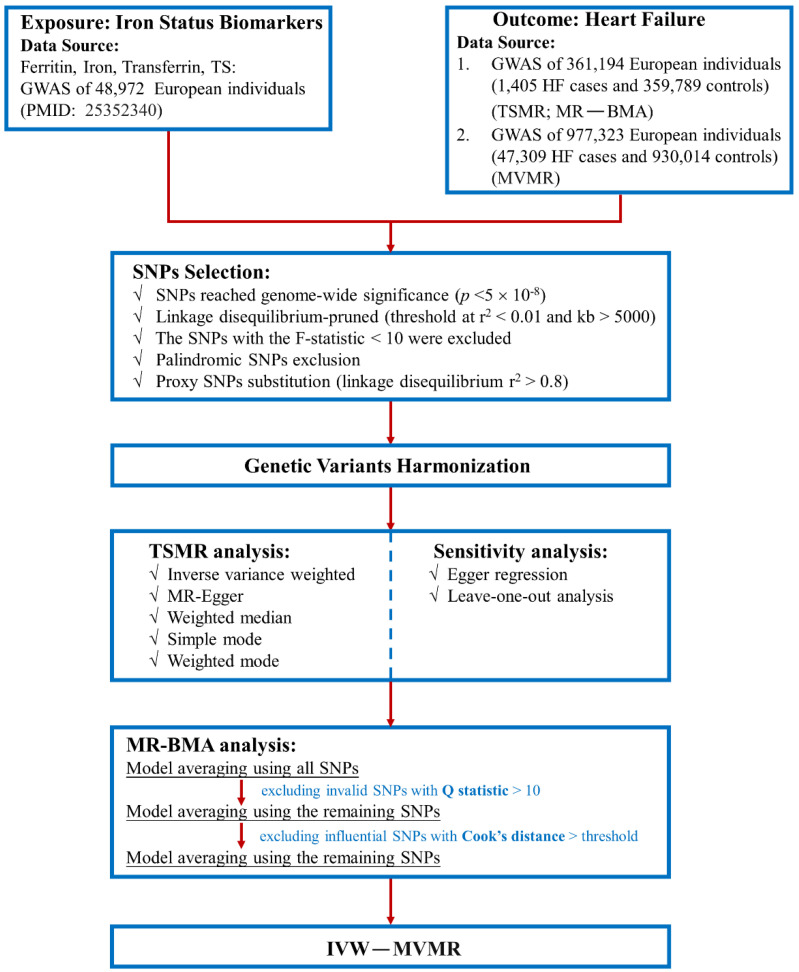
Flowsheet of Mendelian randomisation in this study. TS—transferrin saturation; BMA—Bayesian model averaging.

SNPs associated with iron status markers were extracted from the genome-wide association studies (GWAS) dataset at genome-wide significance thresholds (*p* < 5 × 10^−8^) to obtain powerful IVs. We then searched for the three selected SNPs on PhenoScanner (http://www.phenoscanner.medschl.cam.ac.uk/ (accessed on 17 January 2022)) to identify secondary phenotypes at genome-wide significance (*p* < 5 × 10^−^^8^) [22]. To minimise the correlation between IVs and confounders, we performed linkage disequilibrium (LD) filtering on SNPs with an appropriate threshold (r^2^ < 0.01) [23]. Palindromic SNPs were removed to avoid accidental bias. Subsequently, we harmonised genetic summary data between the remaining SNPs and HF by applying the GWAS HF dataset. When the selected SNP could not be extracted from the HF dataset, we selected SNPs with strong correlations (r^2^ > 0.8) as proxies [24]. Finally, the F-statistics for each SNP were calculated to assess the effect of intensity of the IVs. To minimise potential weak instrument bias, SNPs with F-statistics < 10 were excluded [25]. The screening flowsheet is shown in Figure 2.

### 2.3. MR Estimates

In principal analysis, the fixed-effect inverse variance weighted (IVW) approach was applied to appraise the causal effect of systemic iron status on the risk of HF. Specifically, the Wald estimator and Delta method were employed to generate a causal estimate and standard deviation, respectively, for each IV and, then, the IVW mean of these ratio estimates was calculated as the effect estimate [26]. The results are presented as odds ratios (ORs) for HF risk according to the iron status markers. The ORs were employed to assess the causal effect of iron status parameters on HF if the MR hypotheses were fulfilled [26].

### 2.4. Sensitivity Analysis

The MR-Egger and weighted-median approaches were conducted to determine the reliability of the effect estimate. In the MR-Egger method, the intercept test was performed to estimate the potential horizontal pleiotropy of SNPs. Subsequently, funnel plots were used to visually examine symmetry, which could roughly detect whether causal estimates of weak IVs tended to skew in one direction, and where any bias could indicate potential pleiotropic effects [27]. Cochran’s Q test was applied to evaluate the heterogeneity between estimated Wald ratios for different SNPs. In addition, the weighted-median estimator analysis could provide robust effect estimates if more than half of the IVs in the analysis were valid. Finally, a leave-one-out analysis was performed to determine whether the results were perturbed by a specific SNP [28].

### 2.5. MR-BMA Estimates

To abstain from the limitations of traditional logistic regression methods and to further verify the reliability of the results, we applied a new-style analysis method founded on the Bayesian model averaging (BMA) [29]. The posterior probability (PP) of a specific model and the marginal inclusion probability (MIP) of each iron parameter was calculated, where MIP was defined as the sum of the PPs of all models with risk factors. Moreover, according to the MIP ranking, the model-average causal effect of each iron marker model was generated, which could be used to compare risk factors or to explain the direction of influence. Eventually, the best model was given priority based on the PP value (threshold was 0.02) of the independent model [30]. Q-statistics were employed to check for invalid instruments in the model. Cook’s distance was applied to quantify influential genetic variants [31].

### 2.6. MVMR Analysis

To ascertain the true causal association of iron status biomarkers on HF risk, multivariate MR (MVMR) was conducted (Figure 1b). MVMR can simultaneously detect effects between and with outcomes of multiple risk factors to obtain more accurate estimates of causal effects [32]. The identification of risk factors for HF related to iron status parameters relies mainly on the literature and PhenoScanner database searches. Of note, to ensure the independence of the exposure and outcome samples and to further validate the reliability of our results, another GWAS meta-analysis of data for HF with 47,309 cases and 930,014 controls was applied as an outcome [33]. Summary information on risk factors and HF are presented in Supplementary Table S2. Collinearity of the MVMR model was examined to avoid outcome distortion.

### 2.7. MR Analysis of Diseases with Abnormal Iron Status

We selected two diseases associated with abnormal iron metabolism, hereditary hemochromatosis type 1 (iron overload) and iron deficiency anaemia (ID), to strengthen the integrity of our argument. The GWAS summary statistics for both diseases were obtained from FinnGen (https://www.finngen.fi (accessed on 18 July 2022)), involving 197,405 (including 146 cases and 197,259 controls) and 217,202 (including 6087 cases and 211,115 controls) Europeans, respectively. HF data was provided by the Neale lab analysis from the UK Biobank. Two-sample MR and sensitivity analyses were conducted.

All analyses were performed using the “TwoSampleMR” package (version 0.5.5) in the software R (version 4.0.3; R Foundation for Statistical Computing, Vienna, Austria). A *p*-value less than 0.05 was considered statistically significant.

## 3. Results

### 3.1. SNP Selection and Validation

Collectively, 12 SNPs of iron biomarkers were extracted at a genome-wide significant threshold from the corresponding datasets. Among them, rs1800562 and rs1799945 of the HFE gene and rs855791 of the TMPRSS6 gene were primarily analysed because they were related to all four iron biomarkers. The F-statistic of all SNPs exceeded 10. No SNPs were excluded due to palindromes or linkage disequilibrium (LD) (although both rs1800562 and rs1799945 were located in the HFE gene, their corresponding sites were different (LD r^2^ < 0.01)). The characteristics of these SNPs and their relationships with iron status and HF risk are shown in Table 1.

A search of the PhenoScanner database revealed that all three SNPs were associated with HbA1c [34,35]. In addition, all three SNPs were also associated with erythrocyte traits due to altered iron status. The elevated iron status allele of rs1800562 in the HFE gene was associated with lower low-density lipoprotein levels [36], whereas the elevated iron status alleles of rs1799945 and rs1800562 in the HFE gene were associated with higher diastolic blood pressure [37,38].

### 3.2. Analysis Using the Two-Sample MR

**Figure 3:**
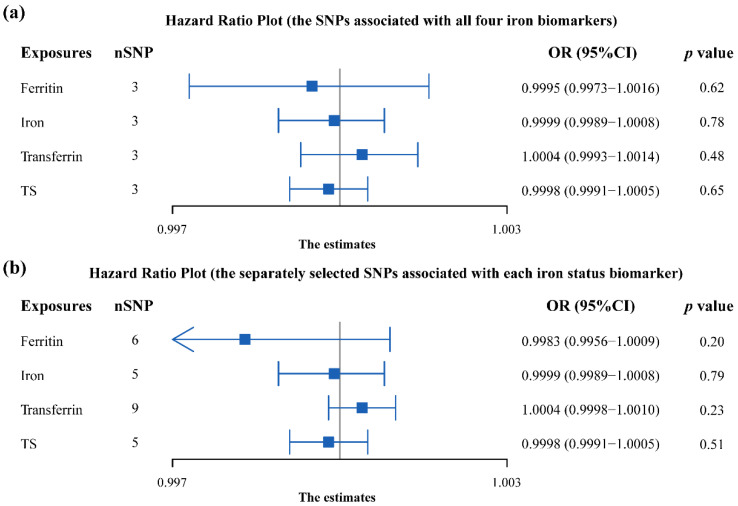
Forest plot summarising the overall Mendelian randomisation estimates of SNP specificity and the causal effect on heart failure. (**a**) Using three SNPs associated with all four iron biomarkers; (**b**) using separately selected SNPs associated with each iron status biomarker. OR—odds ratio; CI—confidence interval.

Results of the MR analysis were expressed as ORs of HF per standard deviation (SD) increase in each iron biomarker, as shown in Figure 3, indicating that there were no causal associations between the four iron biomarker levels and HF (all *p* > 0.05). Consistently, similar results were observed upon employing the fixed-IVW approach and various other statistical methods (Supplementary Figure S3, Supplementary Tables S5 and S6). Results of the scatter plot for each outcome of interest are listed in Supplementary Figure S2.

### 3.3. Sensitivity Analysis

**Figure 4:**
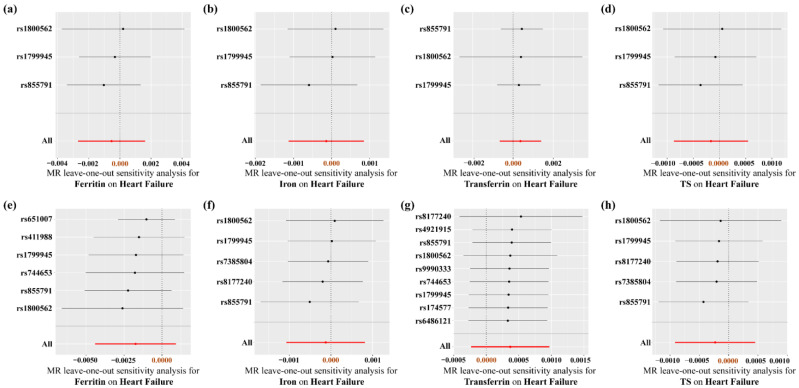
Leave-one-out analysis of the causal association between iron status and heart failure risk. (**a**–**d**) Using three SNPs associated with all four iron biomarkers; (**e**–**h**) using separately selected SNPs associated with each iron status biomarker.

As listed in Supplementary Table S3, there was no heterogeneity in the biomarkers of iron. Moreover, the MR-Egger regression and the appearance of the funnel plots showed that there was a low likelihood of horizontal pleiotropy for our estimations (all *p*-values for MR-Egger intercept > 0.05) (Supplementary Table S4, Supplementary Figure S1). Visually, the leave-one-out analysis plot proved that the results were not altered by the removing of any SNP and were quite robust (Figure 4).

### 3.4. Analysis Using the MR-BMA

Considering pleiotropy, to further test the robustness of the results, we employed MR- BMA analysis to evaluate the causal effect between iron status markers and HF. The results of the MR-BMA are presented in Table 2. We initially included all 12 SNPs. Subsequently, we excluded rs651007 with a Q-statistic value > 10 and included the remaining 11 SNPs. Finally, we excluded rs1800562, whose Cook’s distance exceeded the threshold, and included the remaining 10 SNPs for analysis. Notably, the best model-specific PPs and MIPs for risk factors were compatible with the results from traditional MR methods (all *p* > 0.05). The results of Q-statistic and Cook’s distance are shown in Supplementary Figures S4 and S5, respectively (red boxes represent genes corresponding to invalid and influential instruments).

### 3.5. Analysis Using the MVMR

As shown in Supplementary Table S2, four risk factors that had strong associations with iron status biomarkers, and HF risk were included in the analysis. After adjusting for the risk factors, we still found no causal link between iron status biomarkers and HF (all *p* > 0.05) (Table 3). We also observed a causal association between coronary heart disease and diastolic blood pressure and HF.

### 3.6. MR Analysis of Diseases with Abnormal Iron Status

Characteristics and summary data of the relationship between SNPs and diseases and HF are shown in Supplementary Table S7. There was still insufficient evidence for a direct causal association between these two disturbances of iron metabolism and the risk of HF. Sensitivity analysis showed that the results were robust. (Supplementary Tables S8 and S9 and Supplementary Figure S6).

## 4. Discussion

We examined the causal relationship between four biomarkers of systemic iron status parameters and HF applying MR analysis. We further evaluated the robustness of the results employing MR-BMA and MVMR analysis. Overall, we did not find evidence that these iron state biomarkers were causally associated with HF risk, at least from a genetic perspective.

Iron is essential for numerous biological processes [6]. Although iron status is associated with HF, the evidence for this is mixed. Previous studies have suggested that abnormal iron status (iron overload or deficiency) is associated with a variety of cardiovascular diseases and their risk factors [14,39,40,41,42]. A two-sample MR study revealed that a higher iron status was protective against coronary heart disease [39]. Several cross-sectional clinical studies have demonstrated that serum ferritin and transferrin are positively associated with the risk of hypertension [40,41]. Moreover, recent genetic evidence supported a causal relationship between anomalous systemic iron status and an increased risk of type 2 diabetes [42]. Notably, several cohort studies suggested that iron imbalance is associated with an increased risk of HF [14,15]. However, the findings of this study regarding the influence of iron status biomarkers on HF differ from those of previous studies. One reason may be that in observational studies, confounding factors are unavoidable, which may lead to biased results. In addition, the small sample size of some traditional observational studies may also affect the reliability of the results.

Observational studies are often at risk of confounders, which can mask the true results [43]. Determining causality, through randomised controlled trials (RCTs), can provide solid and effective evidence [44]. However, with regard to complex diseases such as cardiovascular disease, qualified RCTs are expensive, require adequate sample sizes, and require a large number of variables to be controlled [43,44]. Therefore, MR analysis is an alternative method for use. Similar to RCT, the random separation of alleles helps to independently divide the sample into exposure and control groups, with unmeasured confounding factors evenly distributed in both groups [45]. Thus, this effectively eliminates reverse causal or residual confounding effects, leading to the robustness of the results. Furthermore, MR research selects appropriate IVs from publicly available GWAS databases, saving time and reducing cost, and has gradually become a hot topic in epidemiological research in recent years [46].

Hereditary hemochromatosis (HH) is a disorder of iron metabolism that leads to iron overload and damage to multiple organs, including liver, skin, joints, and heart [47]. Among them, type 1 is the most common (the prevalence was 0.4% in the European population) and is associated with the HFE gene [48]. Clinically, the main cardiac manifestations of HH are cardiomyopathy, arrhythmias, and HF. However, to date, robust evidence for the relationship between HH and cardiovascular risk is still lacking and is based only on case reports or small studies. A recent retrospective cohort study showed that HH was strongly associated with the risk of cardiac arrhythmias, but not with HF risk [49]. Combined with our findings, it is suggested that the clinically observed association between HH and HF may be caused by an indirect mechanism.

Regarding HF risk in the general population, our results do not contradict those of previous studies describing the influence of intravenous iron treatments. As a matter of fact, clinical guidelines have recommended the application of intravenous iron treatment to relieve symptoms in HF patients with ID [50]. Recent clinical RCT trials also confirmed the reduction of symptoms and re-hospitalisation risk after intravenous iron therapy in these patients [51,52,53]. However, intravenous iron therapy did not reduce the risk of cardiovascular mortality in acute HF patients with ID [53]. Considering the potential deleterious effects of iron overload [17,54], iron status intervention strategies may not be beneficial when used in the general population or in HF patients without ID.

Moreover, recent studies revealed that systemic iron status is not equivalent to cardiac iron status [55,56,57]. An endocardial biopsy specimen study of 80 HF patients demonstrated that cardiac iron was not associated with systemic iron homeostasis [56]. Systemic iron disorder does not directly alter cardiac iron levels. In one rat ID model, systemic ID did not affect cardiac ID, regardless of whether the heart was normal or failing [55]. Contrarily, in another rat model, iron repletion only led to a slight increase in cardiac iron [57]. These findings indicated that cardiac iron levels may have some resistance to systemic iron disorders, which provides a plausible explanation for why systemic ID does not affect the pathogenesis of HF.

Ferroptosis, which differs from apoptosis, thanatosis, and other types of cell death, is a form of iron-dependent cell death. It has recently been found to induce myocardial injury and is of vital importance in a great number of cardiovascular diseases [58,59]. The interaction between dissociated intracellular iron overload and hydrogen peroxide via the Fenton reaction causes lipid peroxidation of the polyunsaturated fatty acids constituting biofilms, which in turn initiates iron death [60]. Interestingly, Fang et al., [61] showed that when heart-specific FTH gene (ferritin heavy chain) knockout mice were given a high-iron diet, they experienced severe cardiac injury and cardiac hypertrophy with typical ferroptosis characteristics. However, as mentioned above, systemic iron status has weak effects on myocardial iron homeostasis. Furthermore, the effects of gene-specific knockouts in animals are distinct from SNPs in humans; the former artificially creates an extreme situation. Therefore, for the general population, it may be difficult for systemic iron status to cause myocardial injury through the ferroptosis pathway.

Our study has several limitations. First, since individual data were not available and we only applied summary statistics, the HF data applied in this study were not stratified by subtype or severity. In fact, iron status markers may be more strongly associated with a specific subtype of HF. Hence, further studies are required to explore whether similar results are found among patients with different ethnicities, HF subtypes, and disease severities. Second, other biomarkers related to systemic iron status, such as hepcidin, hemosiderin, and ceruloplasmin, were not included in the analysis owing to the lack of corresponding data in the GWAS. This hindered our investigation of complete macro-regulators of iron metabolism and HF risk in this study. Similarly, the interaction between human biology and the environment on HF risk has been difficult to assess because of the lack of effective exposure statistics related to iron intake. Third, MR also has its limitations. (1) Considering the lifetime effect of SNPs, this study assumes that iron status biomarkers act on HF over a long period of time, which is inconsistent with clinical practice. In addition, for some exposures, the body may have compensatory mechanisms in response to chronically elevated (or reduced) exposure levels. (2) MR cannot easily provide information on acute changes in exposure levels. Perhaps changes to individual long-term average exposure levels could not affect outcomes, but acute responses could. (3) Changes in exposure levels caused by genetic variants are often very modest (most genetic variants explain only 1% to 4% of exposure variation). Finally, in the final analysis, since abnormal iron status disease was used as an exposure, which was dichotomous, we cannot exclude bias due to competing risk factors. Nonetheless, this study provides some clues into the pathophysiologic and therapeutic exploration of HF. We expect that our study will improve awareness regarding the epidemiology and will impact clinical decision making in HF.

## 5. Conclusions

Using a genetic approach, we observed that the systemic iron status is not causally associated with HF risk, indicating that abnormal iron status may not be directly involved in the pathogenesis of HF. The observed strong relationship between abnormal iron status and HF risk may be explained by an indirect mechanism by which abnormal iron status mediates HF risk factors (e.g., coronary heart disease, diastolic pressure). However, further studies based on HF subtype stratification analysis are needed to corroborate our results.

## Figures and Tables

**Table 1:** The characteristics of SNPs and their associations with exposures and HF *.

SNP	Nearest Gene	Chr	Position	EA	EAF	F	SNP-Exposures Association			SNP-HF Association		
							Beta	SE	*p*	Beta	SE	*p*
**Ferritin**												
rs1800562	HFE (C282Y)	6	26,093,141	A	0.067	256	0.204	0.016	1.54 × 10^−38^	−1.74 × 10^−04^	0.00027	0.515
rs1799945	HFE (H63D)	6	26,091,179	C	0.85	53	−0.065	0.01	1.71 × 10^−10^	1.32 × 10^−04^	0.0002	0.511
rs855791	TMPRSS6 (V736A)	22	37,462,936	A	0.446	73	−0.055	0.007	1.38 × 10^−14^	−1.02 × 10^−04^	0.00015	0.486
rs744653	WDR75–SLC40A1	2	1.90 × 10^08^	T	0.854	97	−0.089	0.01	8.37 × 10^−19^	1.35 × 10^−04^	0.00021	0.516
rs651007	ABO	9	1.36 × 10^08^	T	0.202	40	−0.05	0.009	1.31 × 10^−08^	5.88 × 10^−04^	0.00018	0.001
rs411988	TEX14	17	56,709,034	A	0.564	47	−0.044	0.007	1.59 × 10^−10^	1.95 × 10^−04^	0.00015	0.178
**Iron**												
rs1800562	HFE (C282Y)	6	26,093,141	A	0.067	668	0.328	0.016	2.72 × 10^−97^	−1.74 × 10^−04^	0.00027	0.515
rs1799945	HFE (H63D)	6	26,091,179	C	0.85	450	−0.189	0.01	1.10 × 10^−81^	1.32 × 10^−04^	0.0002	0.511
rs855791	TMPRSS6 (V736A)	22	37,462,936	A	0.446	806	−0.181	0.007	1.32 × 10^−139^	−1.02 × 10^−04^	0.00015	0.486
rs8177240	TF	3	1.33 × 10^08^	T	0.669	95	−0.066	0.007	6.65 × 10^−20^	−9.63 × 10^−05^	0.00015	0.526
rs7385804	TFR2	7	1 × 10^08^	A	0.621	95	0.064	0.007	1.36 × 10^−18^	−9.27 × 10^−05^	0.00015	0.533
**Transferrin**												
rs1800562	HFE (C282Y)	6	26,093,141	A	0.067	1446	−0.479	0.016	8.90 × 10^−196^	−1.74 × 10^−04^	0.00027	0.515
rs1799945	HFE (H63D)	6	26,091,179	C	0.85	163	0.114	0.01	9.36 × 10^−30^	1.32 × 10^−04^	0.0002	0.511
rs855791	TMPRSS6 (V736A)	22	37,462,936	A	0.446	47	0.044	0.007	1.98 × 10^−09^	−1.02 × 10^−04^	0.00015	0.486
rs744653	WDR75–SLC40A1	2	1.9 × 10^08^	T	0.854	57	0.068	0.01	1.35 × 10^−11^	1.35 × 10^−04^	0.00021	0.516
rs8177240	TF	3	1.33 × 10^08^	T	0.669	3346	−0.38	0.007	8.43 × 10^−610^	−9.63 × 10^−05^	0.00015	0.526
rs9990333	TFRC	3	1.96 × 10^08^	T	0.46	63	−0.051	0.007	1.95 × 10^−13^	−6.56 × 10^−05^	0.00014	0.651
rs4921915	NAT2	8	18,272,466	A	0.782	104	0.079	0.009	7.05 × 10^−19^	−8.05 × 10^−05^	0.00017	0.643
rs6486121	ARNTL	11	13,355,770	T	0.631	48	−0.046	0.007	3.89 × 10^−10^	−2.03 × 10^−04^	0.00015	0.176
rs174577	FADS2	11	61,604,814	A	0.33	83	0.062	0.007	2.28 × 10^−17^	1.45 × 10^−04^	0.00015	0.338
**TS**												
rs1800562	HFE (C282Y)	6	26,093,141	A	0.067	2127	0.577	0.016	2.19 × 10^−270^	−1.74 × 10^−04^	0.00027	0.515
rs1799945	HFE (H63D)	6	26,091,179	C	0.85	676	−0.231	0.01	5.13 × 10^−109^	1.32 × 10^−04^	0.0002	0.511
rs855791	TMPRSS6 (V736A)	22	37,462,936	A	0.446	889	−0.19	0.008	6.41 × 10^−137^	−1.02 × 10^−04^	0.00015	0.486
rs8177240	TF	3	1.33 × 10^08^	T	0.669	218	0.1	0.008	7.24 × 10^−38^	−9.63 × 10^−05^	0.00015	0.526
rs7385804	TFR2	7	1 × 10^08^	A	0.621	67	0.054	0.008	6.07 × 10^−12^	−9.27 × 10^−05^	0.00015	0.533

* HF data from Neale lab analysis of UK Biobank database. SNP—single nucleotide polymorphisms; HF—heart failure; TS—transferrin saturation; Chr—chromosome; EA—effect alleles; EAF—effect alleles frequency; SE—standard error.

**Table 2:** Evaluation of the causal link between iron status biomarkers and HF applying MR-BMA ^#^.

Risk Factor for Model	Ranking by MIP	MIP	ˆθMACE	Ranking by PP	PP	ˆθλ	*p*
Model averaging employing 12 SNPs							
Ferritin	1	0.771	−0.001	1	0.769	−0.002	0.059
Iron	3	0.079	0	3	0.078	0	0.881
Transferrin	2	0.081	0	2	0.081	0	0.832
TS	4	0.071	0	4	0.07	0	0.941
Model averaging employing 11 SNPs (excluding invalid instrument rs651007 with Q-statistic exceed 10)							
Ferritin	1	0.652	−0.001	1	0.651	−0.001	0.079
Iron	4	0.081	0	4	0.081	0	0.921
Transferrin	2	0.172	0	2	0.172	0	0.337
TS	3	0.096	0	3	0.095	0	0.941
Model averaging employing 10 SNPs (excluding influential instrument rs1800562 with Cook’s distance exceeding the threshold)							
Ferritin	1	0.680	−0.001	1	0.679	−0.002	0.099
Iron	4	0.093	0	4	0.093	0	0.891
Transferrin	2	0.131	0	2	0.131	0	0.475
TS	3	0.097	0	3	0.097	0	0.921

MIP—marginal inclusion probability; MACE—model-average causal effect; MR—Mendelian randomisation; MR-BMA—MR based on Bayesian model averaging; PP—posterior probability; #: all risk factors and the best individual model with a PP value greater than 0.02 are given. A negative causal estimate (ˆθMACE or ˆθλ) represents the protective effect recommended by a model, while a positive value represents a risk factor. ˆθλ is the causal effect estimate of a specific model, ˆθMACE is the average causal effect of a risk factor model.

**Table 3:** Assessing the causal association between iron status and HF using IVW multivariate MR.

Exposures	nSNP	Beta	SE	*p* Value
Iron status biomarkers				
Ferritin	3	0.050	0.134	0.709
Iron	3	2.320	1.429	0.104
Transferrin	8	−0.947	0.587	0.107
Transferrin saturation	3	−2.442	1.493	0.102
Iron status biomarkers and risk factors				
Ferritin	2	−0.080	0.063	0.199
Iron	3	0.312	0.242	0.198
Transferrin	6	−0.134	0.102	0.190
Transferrin saturation	3	−0.310	0.255	0.224
Coronary heart disease	12	0.280	0.027	2.420 × 10^−24^
Diastolic pressure	199	0.022	0.005	1.310 × 10^−05^
Low density lipoprotein	45	0.104	0.064	0.106
HbA1c	6	0.021	0.121	0.864

IVW—inverse variance weighted.

## Data Availability

The data analysed in this study were derived from publicly available databases.

## Supplementary Materials

The following supporting information can be downloaded at: https://www.mdpi.com/article/10.3390/nu14163258/s1. Table S1: characteristics and statistics of studies included based on the GIS consortium study for iron status.; Table S2: characteristics of the SNP summary statistics for exposures and outcome; Table S3: heterogeneity test for each trait; Table S4: summary for directional horizontal pleiotropy tests; Table S5: comparison of the different statistical methods for MR analysis evaluating the causal association between exposures and outcomes; Table S6: MR analysis results of a single SNP; Table S7: the characteristics of SNPs and their associations with abnormal iron status disease and HF; Table S8: comparison of the different statistical methods for MR analysis evaluating the causal association between exposures and outcomes; Table S9: sensitivity analysis for each trait. Figure S1: funnel plot visualizing the SNPs related to iron status; Figure S2: scatter plot visualizing the SNPs related to iron status; Figure S3: MR-Egger and fixed-effect IVW analysis of the causal association between iron status and HF risk; Figure S4: diagnostic plots for invalid SNPs (outliers); Figure S5: diagnostic plots for influential genetic variants; Figure S6: MR and sensitivity analysis of two disorders with iron metabolism.

## References

[1] Crespo-Leiro M.G., Metra M., Lund L.H., Milicic D., Costanzo M.R., Filippatos G., Gustafsson F., Tsui S., Barge-Caballero E., De Jonge N. (2018). Advanced heart failure: A position statement of the Heart Failure Association of the European Society of Cardiology. Eur. J. Heart Fail..

[2] Bauersachs J., de Boer R.A., Lindenfeld J., Bozkurt B. (2022). The year in cardiovascular medicine 2021: Heart failure and cardiomyopathies. Eur. Heart J..

[3] Ziaeian B., Fonarow G.C. (2016). Epidemiology and aetiology of heart failure. Nat. Rev. Cardiol..

[4] Zhang Y., Yuan M., Gong M., Li G., Liu T., Tse G. (2019). Associations Between Prefrailty or Frailty Components and Clinical Outcomes in Heart Failure: A Follow-up Meta-analysis. J. Am. Med. Dir. Assoc..

[5] De Boer R.A., Nayor M., de Filippi C.R., Enserro D., Bhambhani V., Kizer J.R., Blaha M.J., Brouwers F.P., Cushman M., Lima J.A.C. (2018). Association of Cardiovascular Biomarkers With Incident Heart Failure With Preserved and Reduced Ejection Fraction. JAMA Cardiol..

[6] Nazanin Abbaspour R.H., Kelishadi R. (2014). Review on iron and its importance for human health. J. Res. Med. Sci..

[7] Zimmermann M.B., Hurrell R.F. (2007). Nutritional iron deficiency. Lancet.

[8] Jacob C., Altevers J., Barck I., Hardt T., Braun S., Greiner W. (2019). Retrospective analysis into differences in heart failure patients with and without iron deficiency or anaemia. ESC Heart Fail..

[9] Cappellini M.D., Comin-Colet J., de Francisco A., Dignass A., Doehner W., Lam C.S., Macdougall I.C., Rogler G., Camaschella C., Kadir R. (2017). Iron deficiency across chronic inflammatory conditions: International expert opinion on definition, diagnosis, and management. Am. J. Hematol..

[10] Ponikowski P., Jankowska E.A. (2021). Targeting Iron Deficiency in Heart Failure: Existing Evidence and Future Expectations. Circ. Heart Fail..

[11] Kurz K., Lanser L., Seifert M., Kocher F., Polzl G., Weiss G. (2020). Anaemia, iron status, and gender predict the outcome in patients with chronic heart failure. ESC Heart Fail..

[12] Anand I.S., Gupta P. (2018). Anemia and Iron Deficiency in Heart Failure: Current Concepts and Emerging Therapies. Circulation.

[13] Lin F., Huang Y., An D., Zhan Q., Wang P., Lai W., Zeng Q., Dong S., Ren H., Xu D. (2021). Iron deficiency is an independent risk factor of increased myocardial energy expenditure in chronic heart failure patients. Ann. Palliat. Med..

[14] Silvestre O.M., Goncalves A., Nadruz W., Claggett B., Couper D., Eckfeldt J.H., Pankow J.S., Anker S.D., Solomon S.D. (2017). Ferritin levels and risk of heart failure-the Atherosclerosis Risk in Communities Study. Eur. J. Heart Fail..

[15] Klip I.T., Voors A.A., Swinkels D.W., Bakker S.J., Kootstra-Ros J.E., Lam C.S., van der Harst P., van Veldhuisen D.J., van der Meer P. (2017). Serum ferritin and risk for new-onset heart failure and cardiovascular events in the community. Eur. J. Heart Fail..

[16] Dong F., Zhang X., Culver B., Chew H.G., Kelley R.O., Ren J. (2005). Dietary iron deficiency induces ventricular dilation, mitochondrial ultrastructural aberrations and cytochrome c release: Involvement of nitric oxide synthase and protein tyrosine nitration. Clin. Sci..

[17] Das S.K., Wang W., Zhabyeyev P., Basu R., McLean B., Fan D., Parajuli N., DesAulniers J., Patel V.B., Hajjar R.J. (2015). Iron-overload injury and cardiomyopathy in acquired and genetic models is attenuated by resveratrol therapy. Sci. Rep..

[18] Davey Smith G., Hemani G. (2014). Mendelian randomization: Genetic anchors for causal inference in epidemiological studies. Hum. Mol. Genet..

[19] Davies N.M., Holmes M.V., Davey Smith G. (2018). Reading Mendelian randomisation studies: A guide, glossary, and checklist for clinicians. BMJ.

[20] Richmond R.C., Davey Smith G. (2022). Mendelian Randomization: Concepts and Scope. Cold Spring Harb. Perspect. Med..

[21] Benyamin B., Esko T., Ried J.S., Radhakrishnan A., Vermeulen S.H., Traglia M., Gogele M., Anderson D., Broer L., Podmore C. (2014). Novel loci affecting iron homeostasis and their effects in individuals at risk for hemochromatosis. Nat. Commun..

[22] Staley J.R., Blackshaw J., Kamat M.A., Ellis S., Surendran P., Sun B.B., Paul D.S., Freitag D., Burgess S., Danesh J. (2016). PhenoScanner: A database of human genotype-phenotype associations. Bioinformatics.

[23] Machiela M.J., Chanock S.J. (2015). LDlink: A web-based application for exploring population-specific haplotype structure and linking correlated alleles of possible functional variants. Bioinformatics.

[24] Johnson A.D., Handsaker R.E., Pulit S.L., Nizzari M.M., O’Donnell C.J., de Bakker P.I. (2008). SNAP: A web-based tool for identification and annotation of proxy SNPs using HapMap. Bioinformatics.

[25] Burgess S., Thompson S.G. (2013). Use of allele scores as instrumental variables for Mendelian randomization. Int. J. Epidemiol..

[26] Burgess S., Butterworth A., Thompson S.G. (2013). Mendelian randomization analysis with multiple genetic variants using summarized data. Genet. Epidemiol..

[27] Bowden J., Davey Smith G., Burgess S. (2015). Mendelian randomization with invalid instruments: Effect estimation and bias detection through Egger regression. Int. J. Epidemiol..

[28] Burgess S., Thompson S.G. (2017). Interpreting findings from Mendelian randomization using the MR-Egger method. Eur. J. Epidemiol..

[29] Zuber V., Colijn J.M., Klaver C., Burgess S. (2020). Selecting likely causal risk factors from high-throughput experiments using multivariable Mendelian randomization. Nat. Commun..

[30] Efron B., Hastie T., Johnstone I., Tibshirani R. (2004). Least angle regression. Ann. Stat..

[31] Cook R.D. (1979). Influential Observations in Linear Regression. J. Am. Stat. Assoc..

[32] Burgess S., Thompson S.G. (2015). Multivariable Mendelian randomization: The use of pleiotropic genetic variants to estimate causal effects. Am. J. Epidemiol..

[33] Shah S., Henry A., Roselli C., Lin H., Sveinbjornsson G., Fatemifar G., Hedman A.K., Wilk J.B., Morley M.P., Chaffin M.D. (2020). Genome-wide association and Mendelian randomisation analysis provide insights into the pathogenesis of heart failure. Nat. Commun..

[34] Soranzo N., Sanna S., Wheeler E., Gieger C., Radke D., Dupuis J., Bouatia-Naji N., Langenberg C., Prokopenko I., Stolerman E. (2010). Common variants at 10 genomic loci influence hemoglobin A(1)(C) levels via glycemic and nonglycemic pathways. Diabetes.

[35] Wheeler E., Leong A., Liu C.T., Hivert M.F., Strawbridge R.J., Podmore C., Li M., Yao J., Sim X., Hong J. (2017). Impact of common genetic determinants of Hemoglobin A1c on type 2 diabetes risk and diagnosis in ancestrally diverse populations: A transethnic genome-wide meta-analysis. PLoS Med..

[36] Willer C.J., Schmidt E.M., Sengupta S., Peloso G.M., Gustafsson S., Kanoni S., Ganna A., Chen J., Buchkovich M.L., Mora S. (2013). Discovery and refinement of loci associated with lipid levels. Nat. Genet..

[37] Wain L.V., Vaez A., Jansen R., Joehanes R., van der Most P.J., Erzurumluoglu A.M., O’Reilly P.F., Cabrera C.P., Warren H.R., Rose L.M. (2017). Novel Blood Pressure Locus and Gene Discovery Using Genome-Wide Association Study and Expression Data Sets From Blood and the Kidney. Hypertension.

[38] Surendran P., Drenos F., Young R., Warren H., Cook J.P., Manning A.K., Grarup N., Sim X., Barnes D.R., Witkowska K. (2016). Trans-ancestry meta-analyses identify rare and common variants associated with blood pressure and hypertension. Nat. Genet..

[39] Gill D., Del Greco M.F., Walker A.P., Srai S.K.S., Laffan M.A., Minelli C. (2017). The Effect of Iron Status on Risk of Coronary Artery Disease: A Mendelian Randomization Study-Brief Report. Arterioscler. Thromb. Vasc. Biol..

[40] Zhu Y., Chen G., Bo Y., Liu Y. (2019). Markers of iron status, blood pressure and incident hypertension among Chinese adults. Nutr. Metab. Cardiovasc. Dis..

[41] Lee D.H., Kang S.K., Choi W.J., Kwak K.M., Kang D., Lee S.H., Lee J.H. (2018). Association between serum ferritin and hypertension according to the working type in Korean men: The fifth Korean National Health and nutrition examination survey 2010–2012. Ann. Occup. Environ. Med..

[42] Wang X., Fang X., Zheng W., Zhou J., Song Z., Xu M., Min J., Wang F. (2021). Genetic Support of A Causal Relationship Between Iron Status and Type 2 Diabetes: A Mendelian Randomization Study. J. Clin. Endocrinol. Metab..

[43] Lawlor D.A., Harbord R.M., Sterne J.A., Timpson N., Davey Smith G. (2008). Mendelian randomization: Using genes as instruments for making causal inferences in epidemiology. Stat. Med..

[44] Evans D.M., Davey Smith G. (2015). Mendelian Randomization: New Applications in the Coming Age of Hypothesis-Free Causality. Annu. Rev. Genom. Hum. Genet..

[45] Gupta V., Walia G.K., Sachdeva M.P. (2017). ‘Mendelian randomization’: An approach for exploring causal relations in epidemiology. Public Health.

[46] Holmes M.V., Ala-Korpela M., Smith G.D. (2017). Mendelian randomization in cardiometabolic disease: Challenges in evaluating causality. Nat. Rev. Cardiol..

[47] Girelli D., Busti F., Brissot P., Cabantchik I., Muckenthaler M.U., Porto G. (2022). Hemochromatosis classification: Update and recommendations by the BIOIRON Society. Blood.

[48] Hanson E.H., Imperatore G., Burke W. (2001). HFE gene and hereditary hemochromatosis: A HuGE review. Human Genome Epidemiology. Am. J. Epidemiol..

[49] Udani K., Chris-Olaiya A., Ohadugha C., Malik A., Sansbury J., Paari D. (2021). Cardiovascular manifestations in hospitalized patients with hemochromatosis in the United States. Int. J. Cardiol..

[50] Ponikowski P., Voors A.A., Anker S.D., Bueno H., Cleland J.G., Coats A.J., Falk V., Gonzalez-Juanatey J.R., Harjola V.P., Jankowska E.A. (2016). 2016 ESC Guidelines for the diagnosis and treatment of acute and chronic heart failure: The Task Force for the diagnosis and treatment of acute and chronic heart failure of the European Society of Cardiology (ESC). Developed with the special contribution of the Heart Failure Association (HFA) of the ESC. Eur. J. Heart Fail..

[51] Ponikowski P., Kirwan B.-A., Anker S.D., McDonagh T., Dorobantu M., Drozdz J., Fabien V., Filippatos G., Göhring U.M., Keren A. (2020). Ferric carboxymaltose for iron deficiency at discharge after acute heart failure: A multicentre, double-blind, randomised, controlled trial. Lancet.

[52] Martens P., Dupont M., Dauw J., Nijst P., Herbots L., Dendale P., Vandervoort P., Bruckers L., Tang W.H.W., Mullens W. (2021). The effect of intravenous ferric carboxymaltose on cardiac reverse remodelling following cardiac resynchronization therapy-the IRON-CRT trial. Eur. Heart J..

[53] Jankowska E.A., Kirwan B.A., Kosiborod M., Butler J., Anker S.D., McDonagh T., Dorobantu M., Drozdz J., Filippatos G., Keren A. (2021). The effect of intravenous ferric carboxymaltose on health-related quality of life in iron-deficient patients with acute heart failure: The results of the AFFIRM-AHF study. Eur. Heart J..

[54] Kremastinos D.T., Farmakis D. (2011). Iron overload cardiomyopathy in clinical practice. Circulation.

[55] Paterek A., Okninska M., Chajduk E., Polkowska-Motrenko H., Maczewski M., Mackiewicz U. (2021). Systemic iron deficiency does not affect the cardiac iron content and progression of heart failure. J. Mol. Cell. Cardiol..

[56] Hirsch V.G., Tongers J., Bode J., Berliner D., Widder J.D., Escher F., Mutsenko V., Chung B., Rostami F., Guba-Quint A. (2020). Cardiac iron concentration in relation to systemic iron status and disease severity in non-ischaemic heart failure with reduced ejection fraction. Eur. J. Heart Fail..

[57] Paterek A., Kepska M., Sochanowicz B., Chajduk E., Kolodziejczyk J., Polkowska-Motrenko H., Kruszewski M., Leszek P., Mackiewicz U., Maczewski M. (2018). Beneficial effects of intravenous iron therapy in a rat model of heart failure with preserved systemic iron status but depleted intracellular cardiac stores. Sci. Rep..

[58] Tang D., Kang R., Berghe T.V., Vandenabeele P., Kroemer G. (2019). The molecular machinery of regulated cell death. Cell Res..

[59] Wu X., Li Y., Zhang S., Zhou X. (2021). Ferroptosis as a novel therapeutic target for cardiovascular disease. Theranostics.

[60] Stockwell B.R., Friedmann Angeli J.P., Bayir H., Bush A.I., Conrad M., Dixon S.J., Fulda S., Gascon S., Hatzios S.K., Kagan V.E. (2017). Ferroptosis: A Regulated Cell Death Nexus Linking Metabolism, Redox Biology, and Disease. Cell.

[61] Fang X., Cai Z., Wang H., Han D., Cheng Q., Zhang P., Gao F., Yu Y., Song Z., Wu Q. (2020). Loss of Cardiac Ferritin H Facilitates Cardiomyopathy via Slc7a11-Mediated Ferroptosis. Circ. Res..

