# Supplementary material for: Appraising the Causal Association between Systemic Iron Status and Heart Failure Risk: A Mendelian Randomisation Study
Source: Nutrients. 2022 Aug 9;14(16):3258. doi: 10.3390/nu14163258 (PMC9412602; doi:10.3390/nu14163258)
Supplement: Supplementary file 1 [file nutrients-14-03258-s001.zip › Supplementary Materials.pdf]

nutrients

Supplemental Materials

Table S1. Characteristics and statistics of studies included based on the GIS consortium study for iron status.

Cohort	Full name	PMID	Gender	N*	Age (years)	Cohort Statistics, Mean ± SD			
						Iron(μmol/l)	Transferrin(g/l)	Saturation (%)	Ferritin (log10, (μg/l)
**Discovery Cohorts:**									
Australia Adult	QIMR Berghofer Adult	19820699; 21151130; 20802479.	M	3432	47.5±12.3	21.2±6.4	2.7±0.35	32.1±10.5	2.30±0.34
			F	5716	46.0±12.8	18.5±6.7	2.9±0.47	26.7±10.4	1.82±0.41
Australia Adolescent	QIMR Berghofer Adolescent	17539372; 22563384	M	1230	14.6±2.0	17.3±5.5	2.9±0.36	24.0±8.22	1.70±0.23
			F	1314	14.9±2.3	16.3±5.4	3.0±0.38	22.3±7.7	1.56±0.26
Estonia (original)	Estonian Genome Project	24518929	M	440	37.3±15.4	19.4±7.7	2.7±0.44	29.9±12.5	1.99±0.39
			F	453	37.5±15.7	16.9±7.4	2.9±0.58	24.0±11.7	1.51±0.44
Val Borbera	Val Borbera Study		M	733	54.4±18.4	17.7±6.3	2.4±0.4	29.6±11.4	1.9±0.4
			F	926	54.8±18.7	16.4±5.8	2.5±0.5	26.9±10.5	1.8±0.4
NBS	Nijmegen Biomedical Study		M	889	66.3±7.1	18.3±5.8	N/A	32.0±11.0	2.19±0.36
			F	902	56.6±10.8	16.3±5.5	N/A	27.5±10.0	1.87±0.39
Cambridge	UK Blood Services (UKBS) Common Controls panel	17554300	M	1198	45.1±11.9	N/A	N/A	N/A	3.29±0.73
			F	1221	42.1±12.7	N/A	N/A	N/A	2.69±0.76
Micros/EIRAC			M	528	45.5±15.8	20.3±7.3	2.60±0.34	31.8±12.4	2.08±0.41
			F	690	46.0±16.7	18.0±7.4	2.79±0.48	26.4±11.2	1.53±0.43
ERF/Rotterdam		15054401; 16877869	M	342	54.6±14.1	21.3±7.0	N/A	36.1±13.4	5.16±0.77
			F	529	52.8±15.1	18.8±6.5	N/A	31.0±11.9	4.23±0.92
KORA F3	Kooperative Gesundheitsforschung in der Region Augsburg	16032513; 16032514	M	809	63.0±10.1	17.5±5.5	2.45±0.33	28.9±9.5	2.33±0.35
			F	825	62.1±10.1	16.1±5.2	2.57±0.36	25.4±8.7	2.00±0.39
KORA F4	Kooperative Gesundheitsforschung	16032513; 16032514	M	882	61.2±8.9	22.2±6.9	2.52±0.36	35.7±12.4	2.31±0.37
			F	927	60.6±8.8	20.2±6.5	2.55±0.35	32.2±11.1	1.97±0.40

	in der Region Augsburg								
BHS	Busselton Health Study		M	397	54.0±15.4	18.6±5.7	2.59±0.21	29.5±10.0	2.20±0.37
			F	480	55.5±14.9	17.1±5.8	2.69±0.50	26.6±11.4	1.81±0.43
**Replication Cohorts:**									
Estonia (replication)	Estonian Genome Project		M	547	54.4±16.1	19.0±6.6	2.7±0.40	31.9±11.5	2.11±0.37
			F	470	53.4±15.9	17.3±6.5	2.8±0.51	29.0±12.7	1.74±0.45
InCHIANTI	nCHIANTI study	19880490	M	536	67.1±15.3	15.4±5.0	1.23±0.5	N/A	4.28±1.0
			F	670	69.1±15.6	14.5±4.4	1.25±0.43	N/A	4.26±0.95
SardiNIA	SardiNIA study on aging	16934002	M	2051	43.7±18.1	17.3±6.4	2.96±0.57	N/A	N/A
			F	2643	43.1±17.3	14.8±6.0	3.15±0.65	N/A	N/A
CoLAUS	Cohorte Lausanne	18366642	M	2550	52.9±10.8	18.3±6.1	2.33±0.33	35.69±12.35	2.28±0.35
			F	2869	52.9±10.8	18.3±6.1	2.33±0.33	35.69±12.35	2.28±0.35
PREVEND	Prevention of Renal and Vascular Endstage Disease		M	1875	50.9±12.8	16.5±5.5	2.54±0.37	26.30±8.99	2.12±0.35
			F	1769	48.2±12.0	15.0±5.6	2.64±0.43	23.30±9.51	1.75±0.43
FENLAND	Fenland Study	21248185	M	615	44.5±7.4	20.0±6.4	2.53±0.37	34.78±12.03	N/A
			F	787	45.4±7.2	17.6±6.8	2.65±0.45	29.48±12.68	N/A
NTERACT (cases)	InterAct (cases)	21717116	M	2087	54.7±8.0	18.5±6.2	2.76±0.40	27.37±10.03	2.24±0.39
			F	2251	55.6±8.3	16.1±5.6	2.89±0.45	22.91±8.68	1.89±0.46
INTERACT (subcohort)	InterAct (subcohort)	21717116	M	1816	52.2±9.2	18.3±6.0	2.72±0.38	27.32±9.38	2.12±0.39
			F	3140	51.7±9.6	16.5±5.9	2.82±0.44	23.91±9.34	1.71±0.43

*, Sample size; GIS, the Genetics of Iron Status consortium.

Table S2. Characteristics of the SNP summary statistics for exposures and outcome.

Trait	GWAS ID	First author	Consortium	Sample size	Population
**Exposures**					
Ferritin	ieu-a-1050	Beben Benyamin	GIS	48,972	European
Iron	ieu-a-1049				
Transferrin	ieu-a-1052				
Transferrin saturation	ieu-a-1051				
**Risk Factors**					
Coronary heart disease	ieu-a-7	Nikpay	CARDIoGRAMplusC4D	184,305	European (majority)
Diastolic pressure	ieu-b-39	Evangelou, E	ICBP	757,601	European
Low density lipoprotein	ieu-b-110	Richardson, Tom	UK Biobank	440,546	European
HbA1c	ieu-b-103	Soranzo N	MAGIC	46,368	European
**Outcome**					
Heart Failure (***TSMR & MR-BMA***)	ukb-d-I50	Neale lab	UK Biobank	361,194	European
Heart Failure (***MVMR***)	ebi-a-GCST009541	Shah S	NA	977,323	European

SNP, single nucleotide polymorphisms; CARDIoGRAMplusC4D, the Coronary Artery Disease Genome wide Replication and Meta-analysis (CARDIoGRAM) plus The Coronary Artery Disease (C4D) Genetics consortium; ICBP, International Consortium of Blood Pressure; MAGIC, the Meta-Analyses of Glucose and Insulin-Related Traits Consortium; TSMR, two sample Mendelian randomization; MR-BMA, MR-Bayesian model averaging; MVMR, multivariate MR.

Table S3. Heterogeneity test for each trait.

Method	Ferritin		Iron		Transferrin		Transferrin saturation	
	Q	Q_*p*val	Q	Q_*p*val	Q	Q_*p*val	Q	Q_*p*val
**Three SNPs associated with all four iron biomarkers**								
MR-Egger	0.7820868	0.3765037	0.8023938	0.3703786	0.6263483	0.4286979	0.7185681	0.396614
Inverse variance weighted	1.1024829	0.576234	1.2655316	0.5311208	0.8546934	0.6522374	1.1386714	0.5659012
**The separately selected SNPs associated with each iron status biomarker**								
MR-Egger	9.0527372	0.0597947	1.9751619	0.5775779	3.3864289	0.8471044	1.6068992	0.6578265
Inverse variance weighted	10.904302	0.0533108	2.0660568	0.7236106	3.90981	0.8651676	1.6981725	0.7910496

Table S4. Summary for directional horizontal pleiotropy tests.

Traits	Ferritin		Iron		Transferrin		Transferrin saturation	
**Three SNPs associated with all four iron biomarkers**								
Egger Regression	Egger intercept	*p*	Egger intercept	*p*	Egger intercept	*p*	Egger intercept	*p*
	0.00011138	0.6720956	0.00029473	0.6195895	-6.85E-05	0.7162109	0.00015075	0.6338951
**The separately selected SNPs associated with each iron status biomarker**								
Egger Regression	Egger intercept	*p*	Egger intercept	*p*	Egger intercept	*p*	Egger intercept	*p*
	-0.0001926	0.4168807	4.46E-05	0.7827327	5.48E-05	0.4928634	-3.64E-05	0.7822986

Table S5. Comparison of the different statistical methods for MR analysis evaluating the causal association between exposures and outcomes.

Methods	Ferritin				Iron				Transferrin				Transferrin saturation			
	N*	Beta	SE	*p*	N	Beta	SE	*p*	N	Beta	SE	*p*	N	Beta	SE	*p*
**Three SNPs associated with all four iron biomarkers**																
MR-Egger		-0.001487	0.002	0.59		-0.001474	0.002	0.60		0.000587	0.0007	0.56		-0.00061	0.0008	0.58
Weighted median		-0.000713	0.0012	0.55		-0.000295	0.0006	0.62		0.000416	0.0005	0.44		-0.000252	0.0004	0.54
Inverse variance weighted	3	-0.000539	0.0011	0.62	3	-0.00014	0.0005	0.78	3	0.000367	0.0005	0.48	3	-0.000163	0.0004	0.65
Simple mode		-0.001338	0.0016	0.49		-0.000616	0.0008	0.52		0.000761	0.0009	0.49		-0.000439	0.0006	0.52
Weighted mode		-0.000912	0.0013	0.57		-0.000589	0.0007	0.50		0.000408	0.0005	0.53		-0.000344	0.0005	0.54
**The separately selected SNPs associated with each iron status biomarker**																
MR-Egger	6	0.000337	0.0027	0.91	5	-0.00037	0.0009	0.72	9	0.000163	0.0004	0.71	5	-9.96E-05	0.0006	0.87
Weighted median		-0.001108	0.0012	0.34		-0.000293	0.0006	0.61		0.000297	0.0003	0.37		-0.000311	0.0004	0.44

Inverse variance weighted	-0.001744	0.0014	0.20	-0.000126	0.0005	0.79	0.000367	0.0003	0.23	-0.00023	0.0003	0.51
Simple mode	-0.001559	0.0015	0.34	-0.000666	0.0008	0.44	0.001153	0.0007	0.12	-0.000586	0.0006	0.37
Weighted mode	-0.00105	0.0012	0.42	-0.00029	0.0007	0.69	0.000305	0.0003	0.38	-0.000348	0.0004	0.47

*: Number of SNPs.

Table S6. MR analysis results of a single SNP.

Traits	Beta	SE	*p value*
**Ferritin_3***			
rs1799945	-0.00203	0.003094	0.510918
rs1800562	-0.00086	0.001313	0.514753
rs855791	0.001846	0.002649	0.485751
All - Inverse variance weighted	-0.00054	0.001099	0.624191
All - MR Egger	-0.00149	0.002004	0.593594
**Ferritin**			
rs1799945	-0.00203	0.003094	0.510918
rs1800562	-0.00086	0.001313	0.514753
rs411988	-0.00444	0.003299	0.178191
rs651007	-0.01177	0.003561	0.000952
rs744653	-0.00152	0.002343	0.516337
rs855791	0.001846	0.002649	0.485751
All - Inverse variance weighted	-0.00174	0.001359	0.19943
All - MR Egger	0.000337	0.002685	0.906216
**Iron_3***			
rs1799945	-0.0007	0.001064	0.510918
rs1800562	-0.00053	0.000816	0.514753
rs855791	0.000561	0.000805	0.485751
All - Inverse variance weighted	-0.00014	0.000505	0.781444
All - MR Egger	-0.00147	0.002024	0.599302
**Iron**			
rs1799945	-0.0007	0.001064	0.510918

rs1800562	-0.00053	0.000816	0.514753
rs7385804	-0.00145	0.002322	0.532914
rs8177240	0.00146	0.002299	0.525512
rs855791	0.000561	0.000805	0.485751
All - Inverse variance weighted	-0.00013	0.000482	0.793769
All - MR Egger	-0.00037	0.000942	0.720718
**Transferrin_3***			
rs1799945	0.00116	0.001764	0.510918
rs1800562	0.000364	0.000559	0.514753
rs855791	-0.00231	0.003311	0.485751
All - Inverse variance weighted	0.000367	0.000526	0.484906
All - MR Egger	0.000587	0.000698	0.555053
**Transferrin**			
rs174577	0.002331	0.002432	0.337862
rs1799945	0.00116	0.001764	0.510918
rs1800562	0.000364	0.000559	0.514753
rs4921915	-0.00102	0.002197	0.64277
rs6486121	0.004413	0.003261	0.175949
rs744653	0.00199	0.003066	0.516337
rs8177240	0.000254	0.000399	0.525512
rs855791	-0.00231	0.003311	0.485751
rs9990333	0.001286	0.002843	0.650932
All - Inverse variance weighted	0.000367	0.000307	0.232277
All - MR Egger	0.000163	0.000417	0.707982
**Transferrin Saturation_3***			
rs1799945	-0.00057	0.000871	0.510918
rs1800562	-0.0003	0.000464	0.514753
rs855791	0.000534	0.000767	0.485751
All - Inverse variance weighted	-0.00016	0.000361	0.651645
All - MR Egger	-0.00061	0.000778	0.576839

**Transferrin Saturation**			
rs1799945	-0.00057	0.000871	0.510918
rs1800562	-0.0003	0.000464	0.514753
rs7385804	-0.00172	0.002752	0.532914
rs8177240	-0.00096	0.001517	0.525512
rs855791	0.000534	0.000767	0.485751
All - Inverse variance weighted	-0.00023	0.000349	0.50895
All - MR Egger	-9.96E-05	0.000555	0.869038

*: three SNPs associated with all four iron biomarkers.

Table S7. The characteristics of SNPs and their associations with exposures and HF*.

SNP	Nearest gene	Chr	Position	EA	OA	EAF	SNP-Exposures association			SNP-HF association		
							Beta	SE	p-value	Beta	SE	p-value
**Hereditary hemochromatosis type 1# (GWAS ID: Finn-b-E4_IRON_MET)**												
rs1800562	HFE	6	26093141	A	G	0.037	10.3356	0.5034	1.16E-93	-0.0001744	0.000268	0.514753
rs2029458	GRM4	6	34106536	A	G	0.998	-11.4777	2.0468	2.05E-08	-0.0009129	0.000628	0.146076
rs2744228	/	6	25341405	G	T	0.464	-0.6275	0.1132	3.00E-08	-0.0001299	0.000148	0.380118
rs3094640	HCG17	6	30205643	T	C	0.724	-0.8294	0.1311	2.52E-10	-0.000203	0.000152	0.182267
rs385886	FAM65B	6	24923935	G	A	0.302	0.7414	0.1249	2.94E-09	3.72E-06	0.00016	0.981494
**Iron deficiency anemia (GWAS ID: Finn-b-D3_ANAEMIA_IRONDEF)**												
rs117725035	SMG8	17	57289661	G	A	0.026	0.5924	0.0629	4.63E-21	-0.000699	0.000539	0.194282
rs75362578	BCAS3	17	58819628	G	A	0.025	0.5601	0.065	7.13E-18	0.00017076	0.000374	0.648024
rs78638044	RPS6KB1	17	57974476	A	G	0.083	0.208	0.0343	1.28E-09	0.0003029	0.000216	0.160472

*, HF data from Neale lab analysis of UK Biobank database. #, Feature information in GWAS is "Disorders of iron metabolism", verified by FinGen as "hereditary hemochromatosis type 1" (https://risteys.finngen.fi/endpoint/E4_IRON_MET). SNP, single nucleotide polymorphisms; HF, heart failure; TS, transferrin saturation; Chr, chromosome; EA, effect alleles; EAF, effect alleles frequency; SE, standard error.

Table S8. Comparison of the different statistical methods for MR analysis evaluating the causal association between exposures and outcomes.

Methods	Hereditary hemochromatosis type 1				Iron deficiency anemia			
	N	Beta	SE	*p*	N	Beta	SE	*p*
MR-Egger	5	-1.05E-05	2.86E-05	0.74	3	-1.20E-03	1.30E-03	0.53
Weighted median	5	-1.14E-05	2.49E-05	0.65	3	2.00E-04	6.00E-04	0.77
Inverse variance weighted	5	6.61E-06	2.57E-05	0.80	3	1.00E-04	7.00E-04	0.83
Simple mode	5	2.56E-05	7.30E-05	0.74	3	6.00E-04	8.00E-04	0.56
Weighted mode	5	-6.92E-06	2.52E-05	0.80	3	3.00E-04	7.00E-04	0.74

Table S9. Sensitivity analysis for each trait.

**Methods**	**Hereditary hemochromatosis type 1**		**Iron deficiency anemia**	
**Heterogeneity test**	Q	Q_*p*val	Q	Q_*p*val
MR-Egger	3.430044	0.33	1.608959	0.204638
Inverse variance weighted	5.004318	0.29	3.778578	0.151179
**Pleiotropy tests**	Egger intercept	*p*	Egger intercept	*p*
Egger Regression	0.000121	0.33	0.000563	0.452595

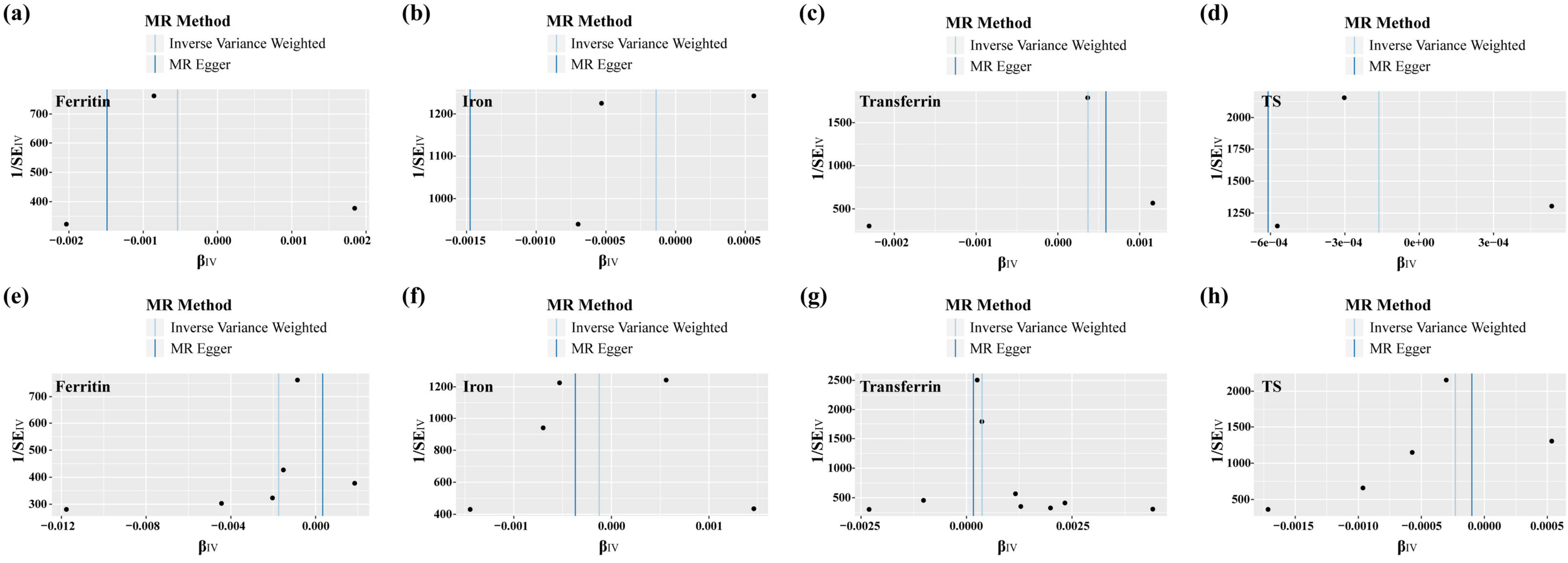

Figure S1. Funnel plot visualizing the SNPs related to iron status. (**a-d**). Using three SNPs associated with all four iron biomarkers; (**e-h**). Using separately selected SNPs associated with each iron status biomarker. Each black dot represented a SNP, with the overall estimate obtained from two methods. Funnel plots were used to visually examine symmetry, which can roughly detect whether causal estimates of weak variants tended to skew in one direction.

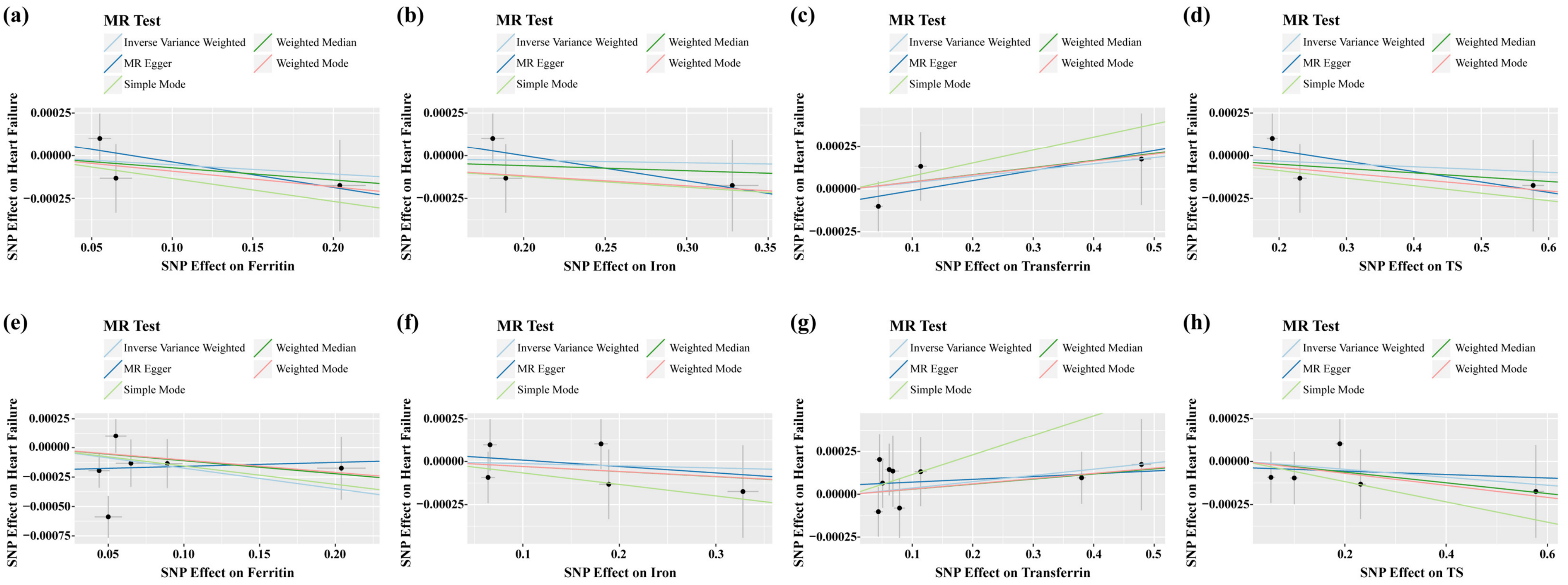

Figure S2. Scatter plot visualizing the SNPs related to iron status. (**a-d**). Using three SNPs associated with all four iron biomarkers; (**e-h**). Using separately selected SNPs associated with each iron status biomarker. Each black dot represents a SNP, plotted by SNP estimates at the systematic iron biomarker level and SNP estimates at the risk of HF individual with standard error bars. The slope of the line corresponds to the causal estimates made using five different methods.

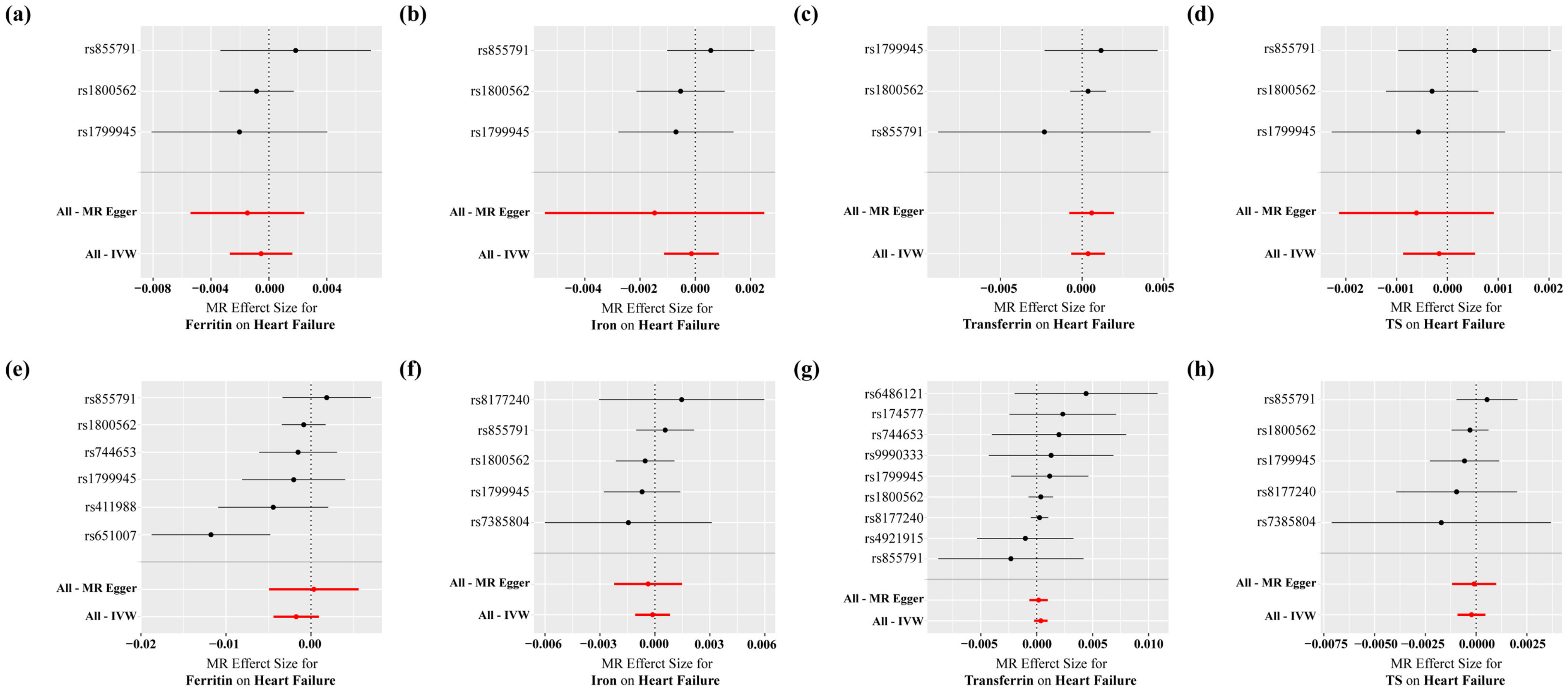

Figure S3. MR Egger and fixed-effect IVW analysis of the causal association between iron status and HF risk. (**a-d**). Using three SNPs associated with all four iron biomarkers; (**e-h**). Using separately selected SNPs associated with each iron status biomarker. Black dots represent the estimated betas, and horizontal lines represent the 95% CI of the betas. Forest plots reflect the causal effect of individual SNPs on heart failure. IVW, inverse variance weighted. CI, confidence interval.

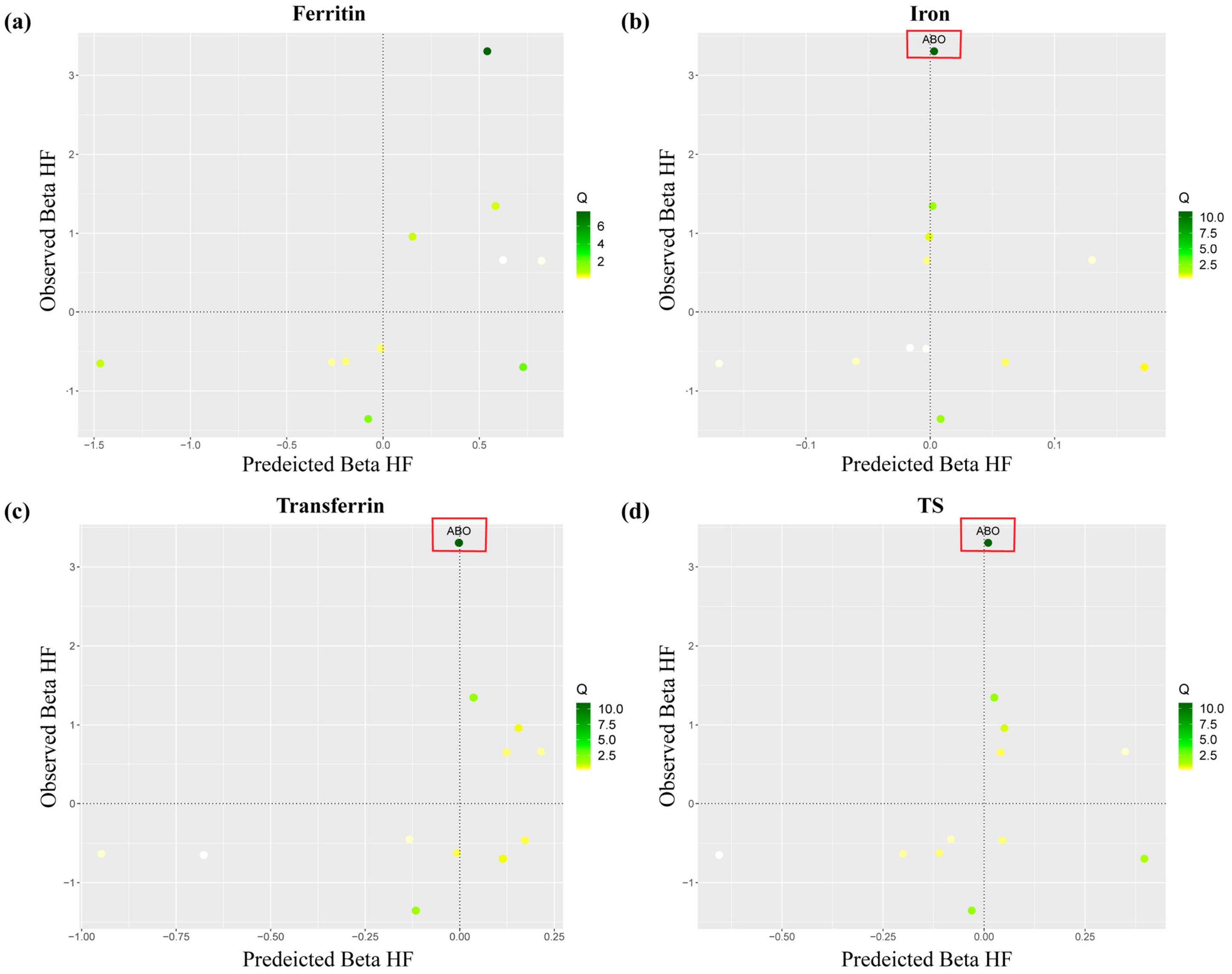

Figure S4. Diagnostic plots for invalid SNPs (outliers). (**a**). Ferritin; (**b**). Iron; (**c**). Transferrin; (**d**). Transferrin saturation. When Q-statistic > 10, the instrument for the corresponding gene is invalid and marked with a red box.

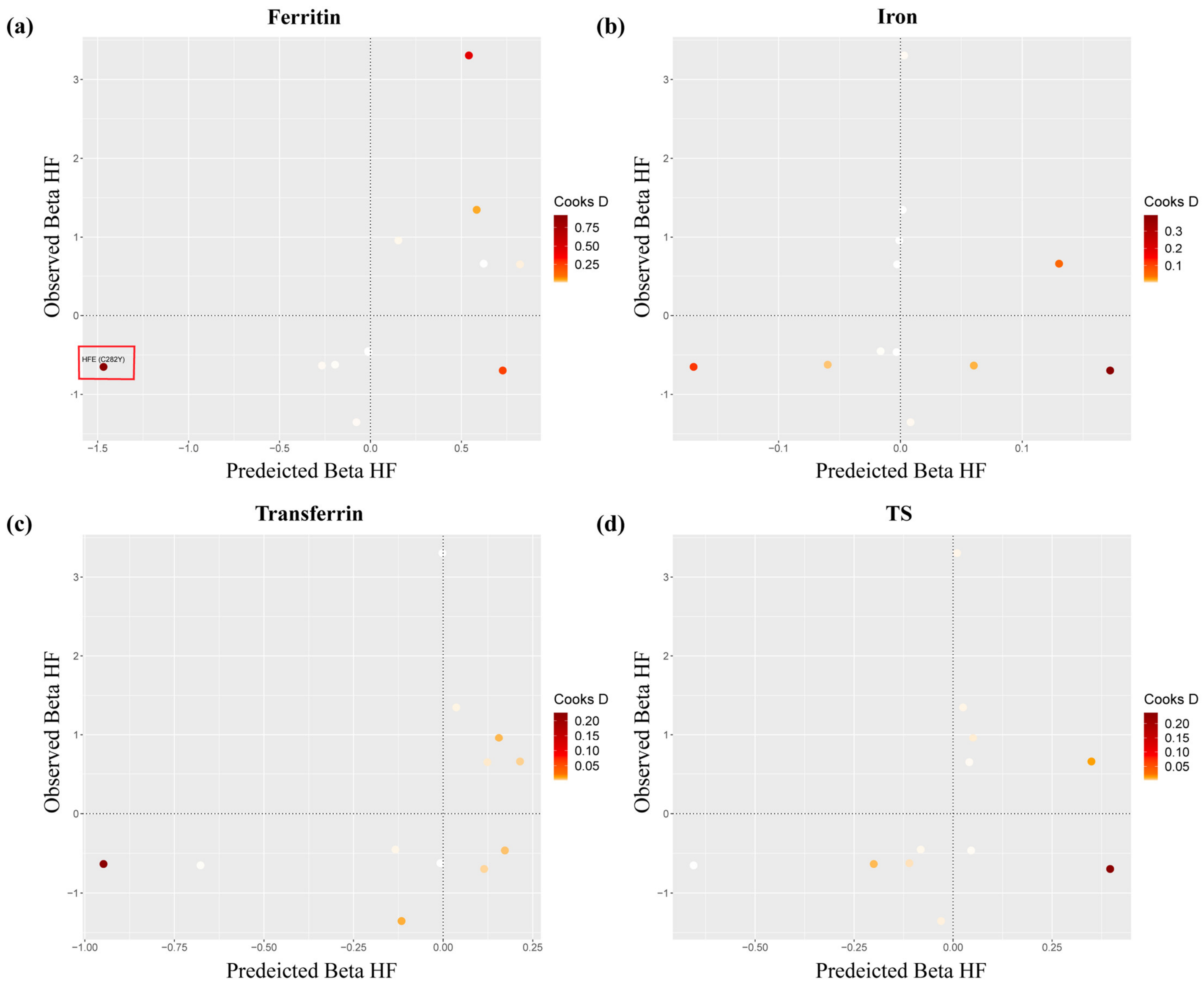

Figure S5. Diagnostic plots for influential genetic variants. (**a**). Ferritin; (**b**). Iron; (**c**). Transferrin; (**d**). Transferrin saturation. When Cook's distance > the threshold, the instrument for the corresponding gene is influential and marked with a red box.

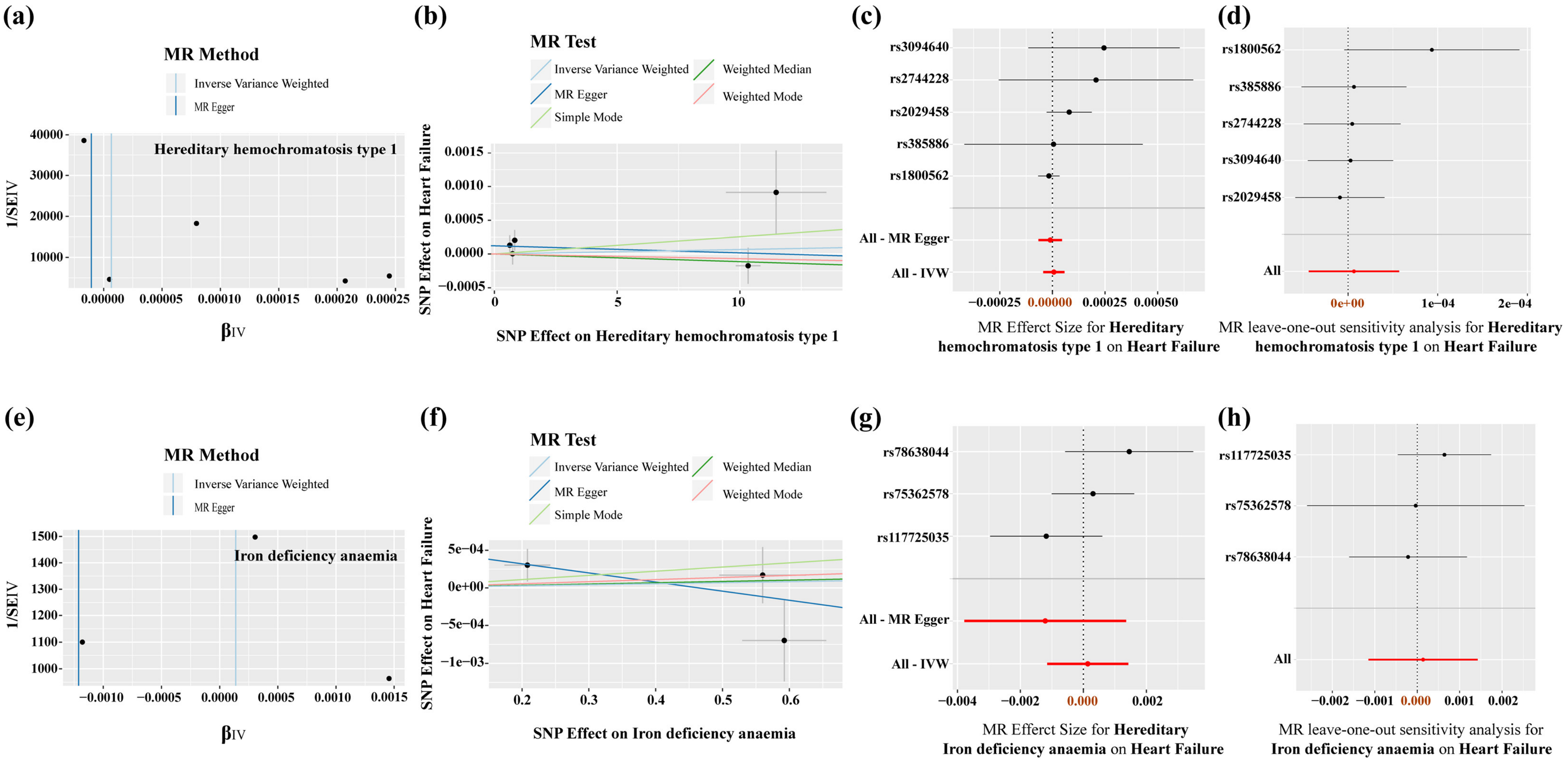

Figure S6. MR and sensitivity analysis of two disorders with iron metabolism. (**a-d**). Hereditary hemochromatosis type 1; (**e-f**). Iron deficiency anemia.
