# Supplementary figures and images for: Appraising the Causal Association between Systemic Iron Status and Heart Failure Risk: A Mendelian Randomisation Study
Source: Nutrients. 2022 Aug 9;14(16):3258. doi: 10.3390/nu14163258 (PMC9412602; doi:10.3390/nu14163258)

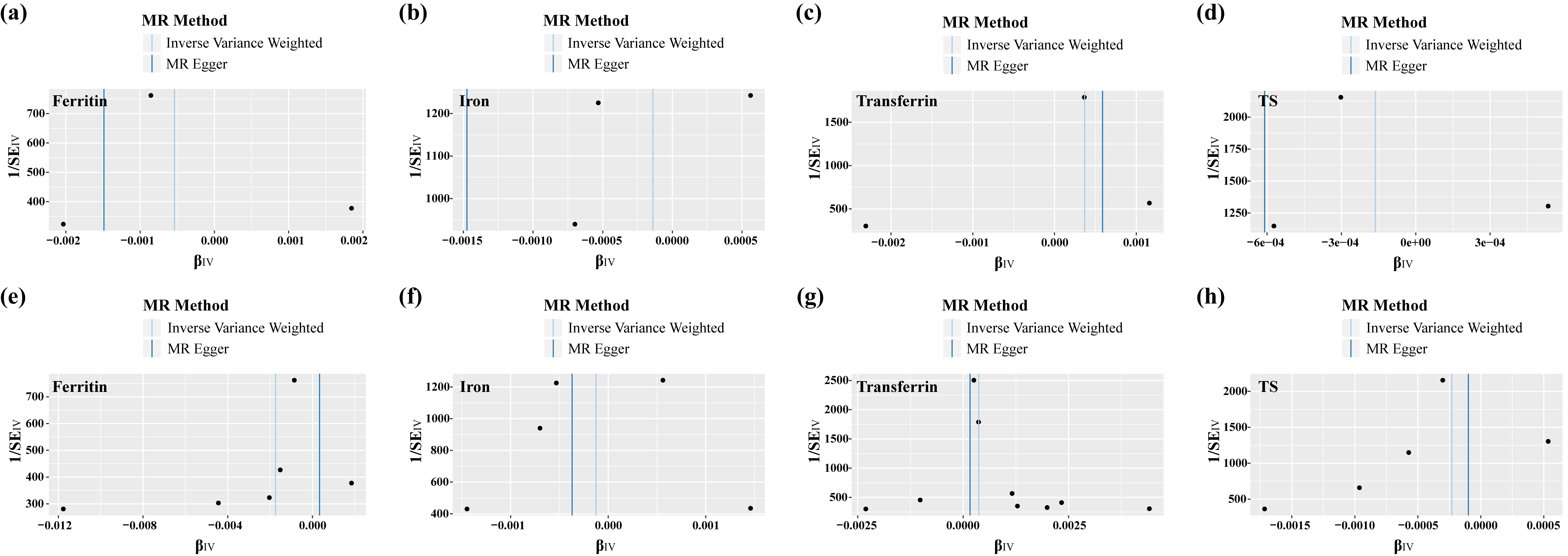

Supplement: Supplementary file 1 [file nutrients-14-03258-s001.zip › Supplementary Figure S1.tif]

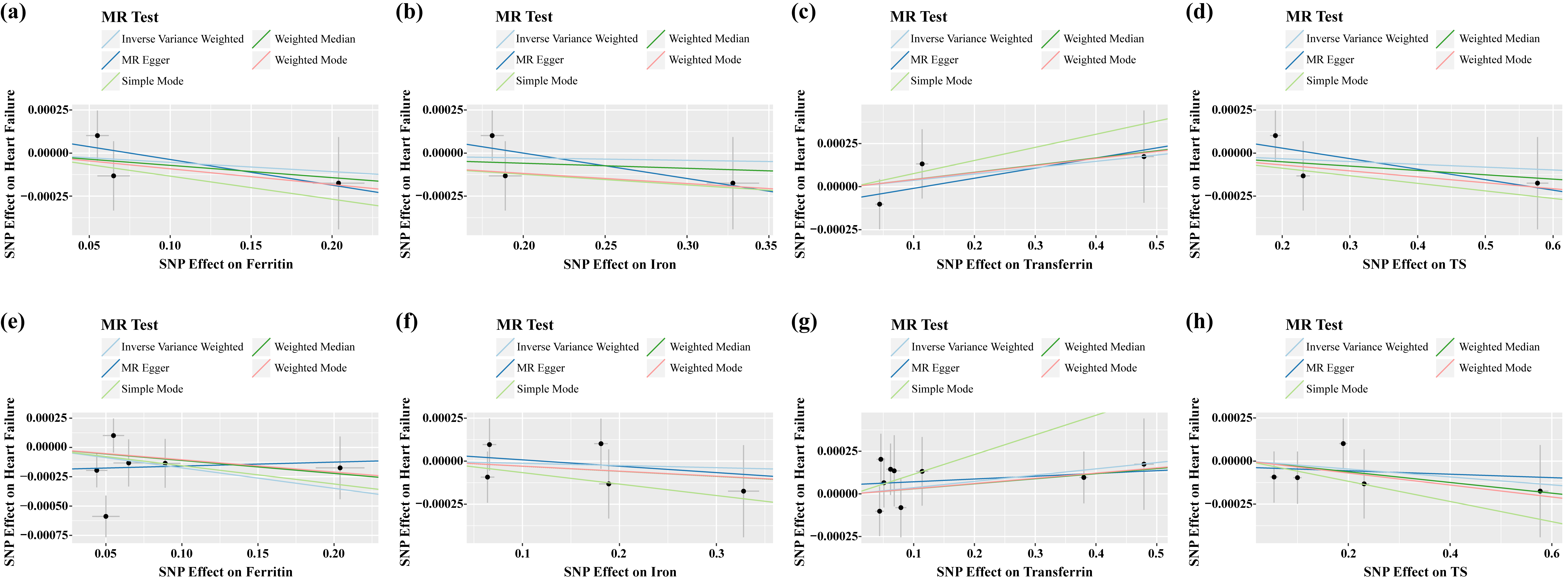

Supplement: Supplementary file 1 [file nutrients-14-03258-s001.zip › Supplementary Figure S2.tif]

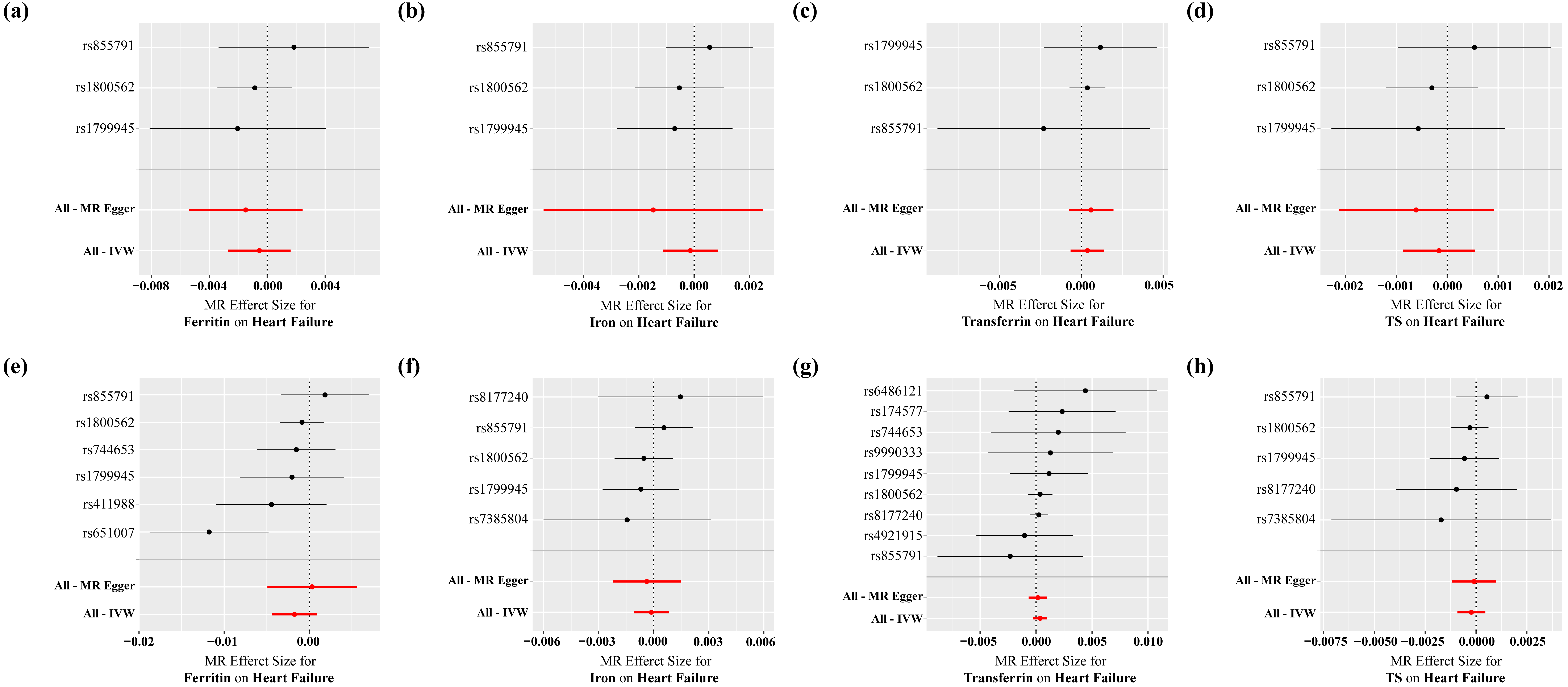

Supplement: Supplementary file 1 [file nutrients-14-03258-s001.zip › Supplementary Figure S3.tif]

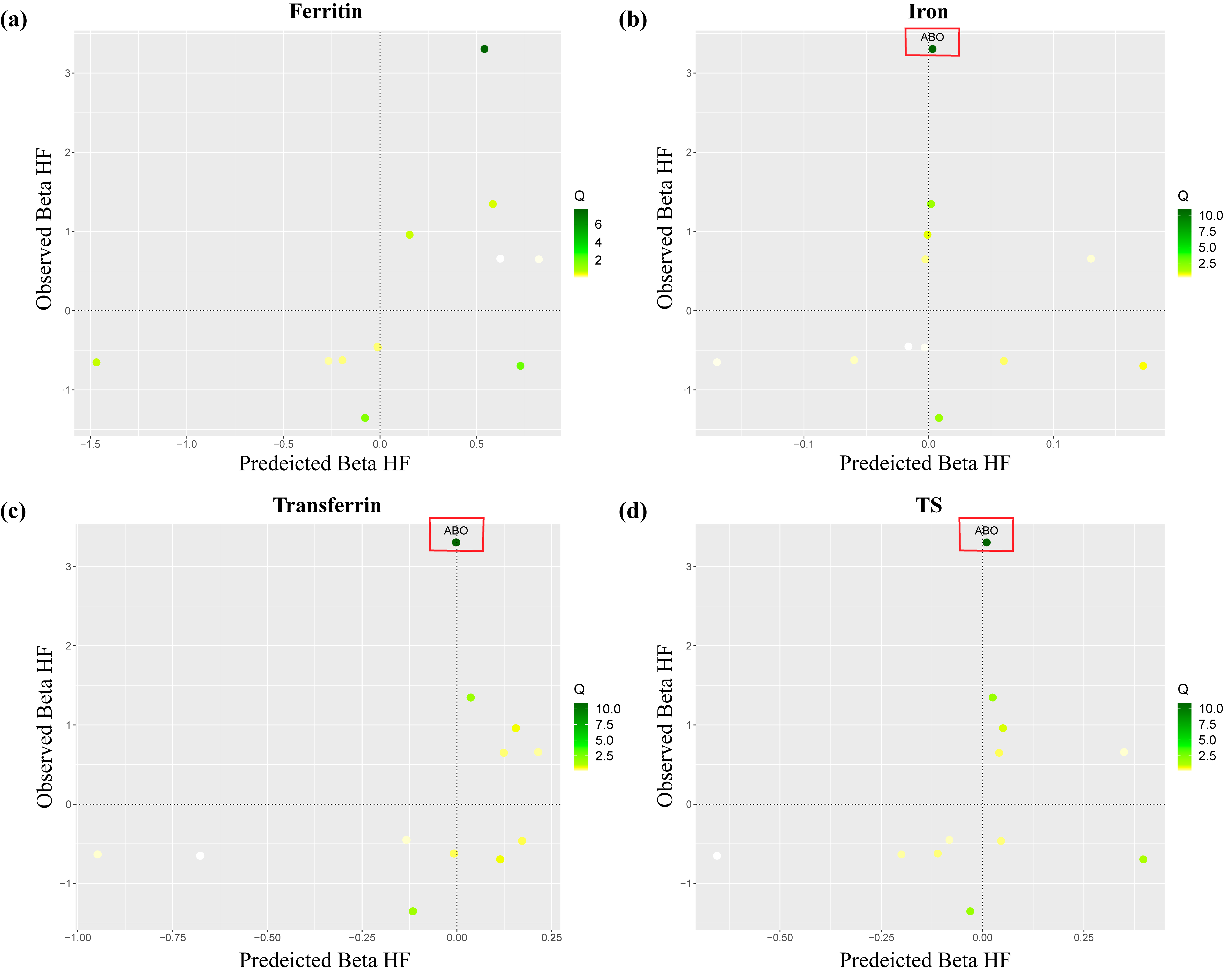

Supplement: Supplementary file 1 [file nutrients-14-03258-s001.zip › Supplementary Figure S4.tif]

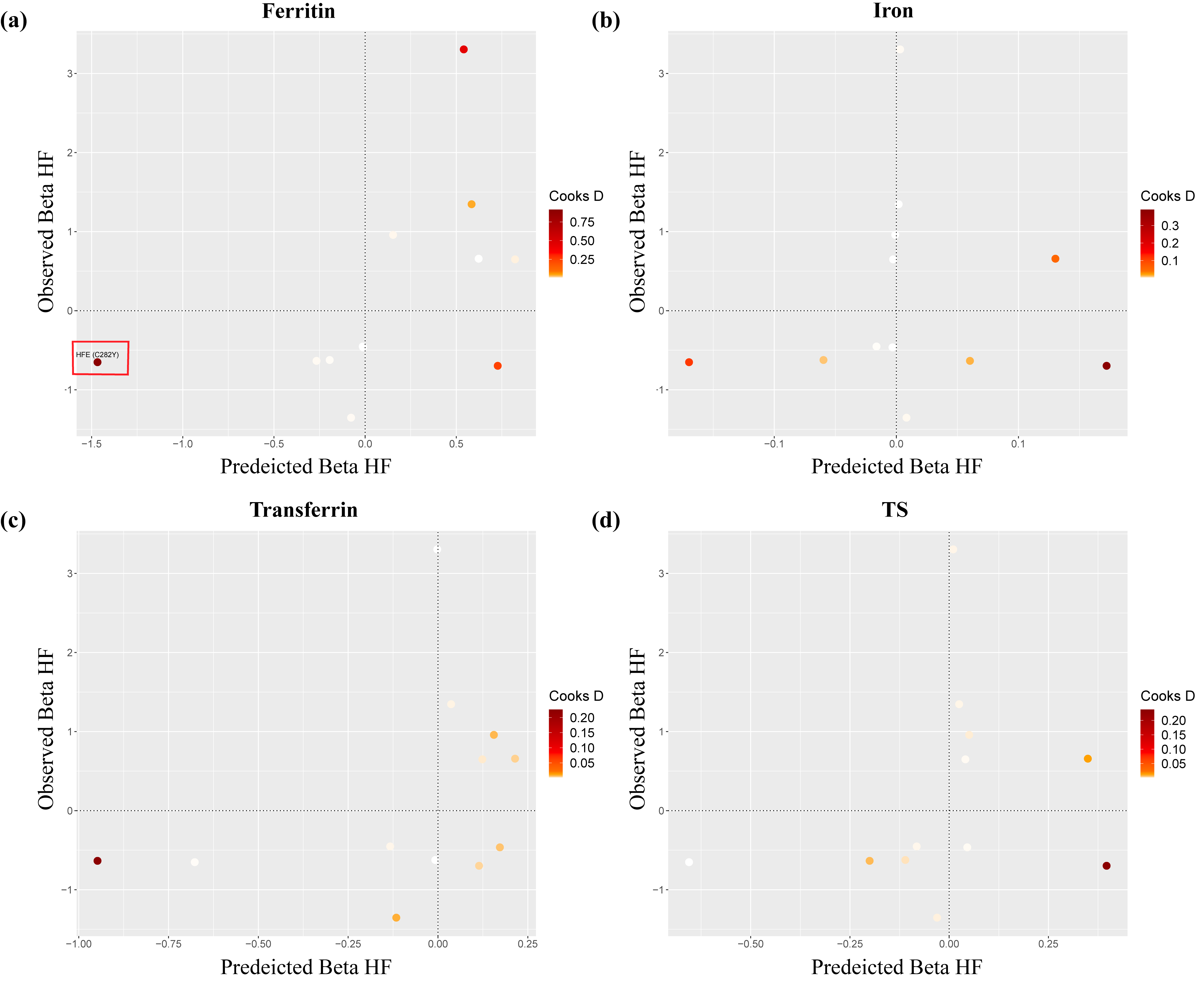

Supplement: Supplementary file 1 [file nutrients-14-03258-s001.zip › Supplementary Figure S5.tif]

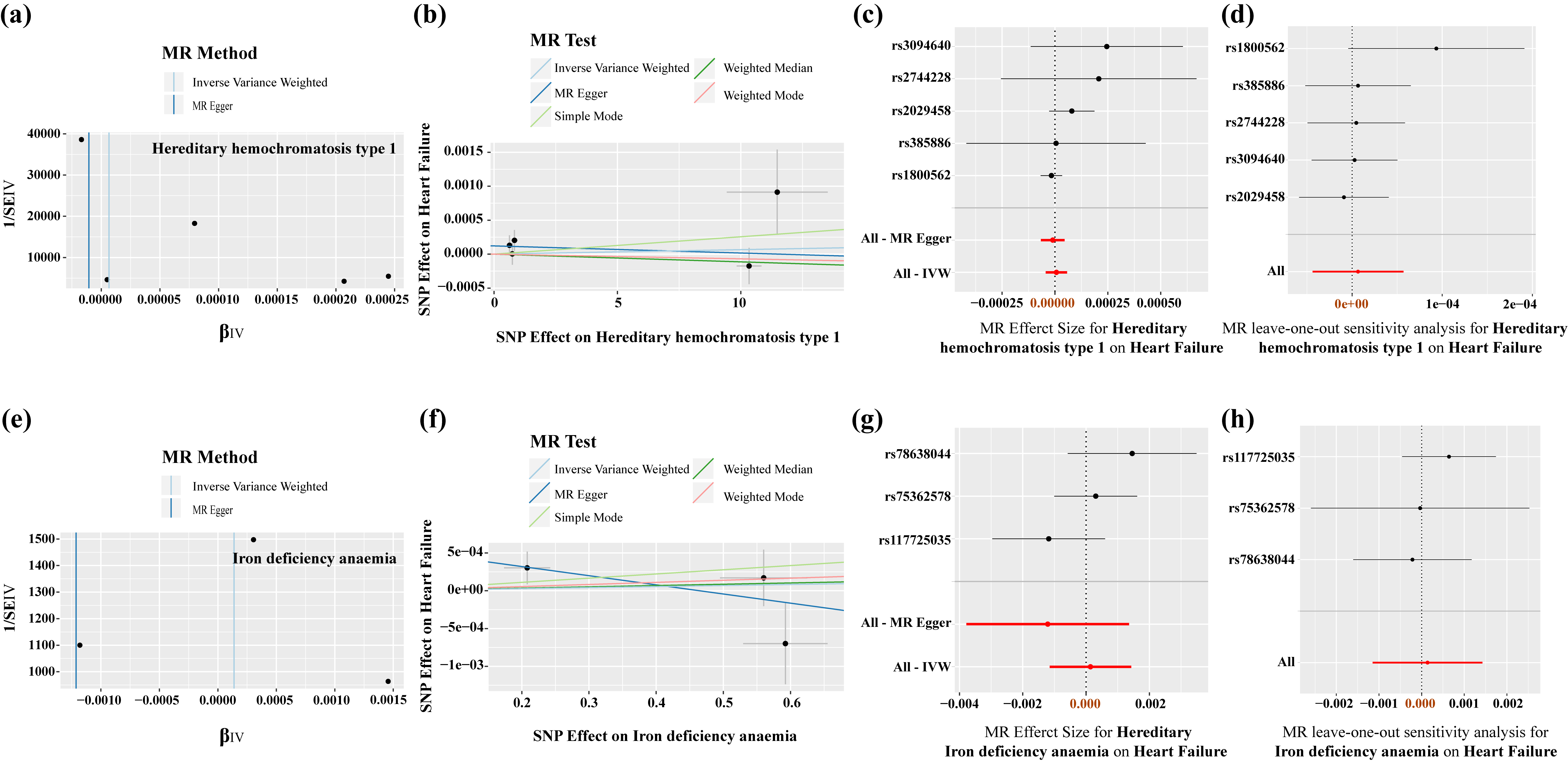

Supplement: Supplementary file 1 [file nutrients-14-03258-s001.zip › Supplementary Figure S6.tif]
